# Comparative Assessment of Wastewater-Based Surveillance Normalization Methods to Improve Pathogen Monitoring in Rural Sewersheds

**DOI:** 10.1021/acs.est.4c14485

**Published:** 2025-05-27

**Authors:** Amanda Darling, Benjamin C. Davis, Thomas Byrne, Madeline Deck, Gabriel E. Maldonado Rivera, Sarah Price, Amber Amaral-Torres, Clayton Markham, Raul A. Gonzalez, Peter J. Vikesland, Leigh-Anne H. Krometis, Amy Pruden, Alasdair Cohen

**Affiliations:** † Department of Population Health Sciences, Virginia Tech, Blacksburg, Virginia 24061, United States; ‡ Department of Civil and Environmental Engineering, 1757 Virginia Tech, Blacksburg, Virginia 24061, United States; § Office of Research and Development, U.S. Environmental Protection Agency, Cincinnati, Ohio 45220, United States; ∥ Genetics, Bioinformatics, and Computational Biology, Virginia Tech, Blacksburg, Virginia 24061, United States; ⊥ Department of Biological Systems Engineering, Virginia Tech, Blacksburg, Virginia 24061, United States; # 143611 Hampton Roads Sanitation District, Virginia Beach, Virginia 23455, United States

**Keywords:** wastewater-based surveillance, normalization, rural health, public health, inflow and infiltration

## Abstract

Many in-sewer dynamics that can impact the fidelity of wastewater-based surveillance results remain understudied. Some conventional approaches for normalizing pathogen signals in sewersheds may not be appropriate when there is substantial inflow and infiltration (I&I). Our objective for this study was to evaluate the effect of multiple normalization approaches on wastewater pathogen signals at the WWTP influent and across a small rural sewershed (<3000 people) with different levels of I&I. We collected wastewater samples monthly, from 2022 to 2023, from the wastewater treatment plant (WWTP) influent and 11 additional sewer system nodes with well-characterized I&I impacts. We quantified concentrations of SARS-CoV-2, norovirus GII, and rotavirus at the WWTP influent and subsewershed sites, and compared normalization approaches using flow, population, physicochemical parameters (COD, TSS, NH_3_(aq), PO_43_^–^–P), and human fecal markers (crAssphage, HF183, mtDNA). Overall, our findings suggest that in systems with substantial I&I, some commonly used normalization approaches, particularly those using physicochemical parameters, may inadvertently introduce additional variability in viral signals measured at WWTP influent and result in wastewater measures that are more closely associated with precipitation trends rather than with pathogen signals. Normalization with human fecal markers appears to be a relatively robust option for sewersheds impacted by substantial I&I.

## Graphical abstract

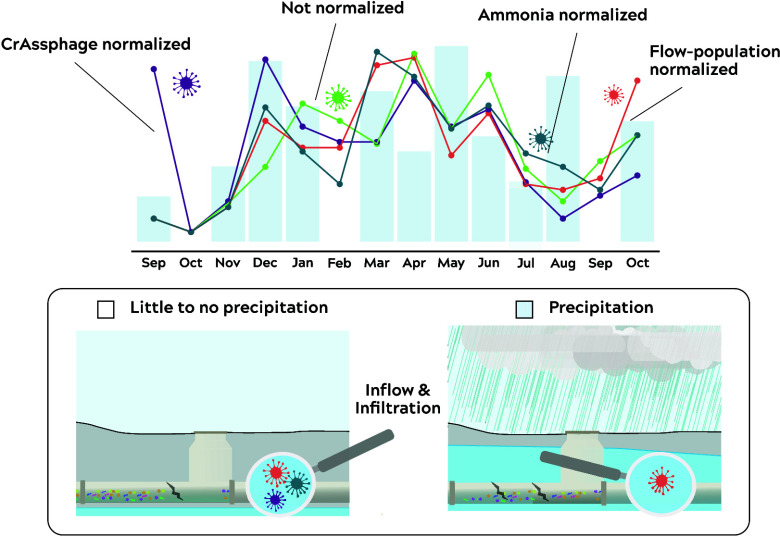

## Introduction

Largely prompted by the COVID-19 pandemic, there has been a rapid expansion of sewage collection and analysis infrastructure to support wastewater-based surveillance (WBS) and wastewater-based epidemiology (WBE) to address various public health data gaps.^1^ Wastewater monitoring offers a number of advantages that can complement the existing clinical testing infrastructure. As a noninvasive form of monitoring population-level community infection patterns, WBS can provide information about both symptomatic and asymptomatic individuals in a much more timely fashion, given that there is often a lag period between fecal shedding or symptom onset.^2^

A challenge of WBS is that signals can change during travel through the sewershed. Sewers not only encompass fecal shedding from households served by a sewer collection network but also comprise distinct environments where various biological, chemical, and physical processes take place.^3^ Within sewer networks, pathogen biomarkers—the metric used to examine population-level patterns of pathogen circulation—may be intermittently or continuously altered by an array of phenomena, including surface water inflow and groundwater infiltration (I&I), decay,^4^ and/or partitioning into biofilms and sediment.^5^

In the context of sewer network functioning, I&I refers to surface or groundwater entering sewer conveyance networks at entry points not intended for such flows.^6^ Inflow, specifically, refers to the entry of surface water or stormwater sewers via poorly sealed manhole covers or storm drains.^6^ Infiltration results from groundwater seeping into sewer systems through cracks in pipes, leaky pipe joints, and deteriorated manholes. Such processes can occur year-round due to elevated water tables or as a result of precipitation events.^7^ I&I are clearly problematic for WBS, resulting in the dilution of wastewater disease signal. Without some form of normalization, wastewater data may not accurately reflect population-level pathogen shedding trends. Several sampling strategies, modeling techniques, and normalization approaches have been suggested to more accurately relate biomarker measures in wastewater to actual pathogen carriage in the corresponding human population.^8–13^ Increased biological replicates and sampling frequency^14,15^ as well as smoothing and forecasting models^16^ can help to account for the inherently variable nature of collection, processing, and quantification of viral nucleic acid. However, it is unclear whether these approaches can address biases introduced in situ, such as precipitation-driven increases in I&I contribution and various other in-sewer phenomena. Thus, there is a need for systematic examination of the efficacy of various approaches for improving the fidelity of WBS signal, especially in settings experiencing substantial I&I.

A number of parameters could potentially be used to normalize pathogen signal in wastewater in a manner that accounts for in situ variability in a sewershed. Biomarkers of human fecal shedding are becoming more widely used as a proxy for the relative strength or dilution of sewage.^17^ Common biomarkers used for this purpose include crAssphage, a bacteriophage highly abundant and specific to human fecal metagenomes,^18,19^ the hCYTB484 gene targeting the human mitochondrial genome, which has exhibited a 100% detection in human feces and 97% specificity to humans compared to animal feces even where relatively fewer individuals contribute to input waste streams,^20^ and HF183, a human-associated *Bacteroides* marker.^21^ Various physicochemical measures of sewage strength are routinely measured by wastewater treatment plants (WWTPs) (e.g., total suspended solids, chemical oxygen demand)^22^ and are also logical proxies for general human inputs to sewage.^23^ In theory, any of these measures of wastewater strength can be applied as a normalization scaling factor to improve estimates of pathogen loadings per capita.^23–27^

Others have proposed normalization approaches to specifically address precipitation-driven dilution of target biomarkers.^28,29^ Bench-scale spiking experiments have indicated that the relative quantification of viral nucleic acid normalized against crAssphage abundance remained stable with increasing levels of dilution.^29^ However, it is not well understood whether the behavior of crAssphage is representative of the behavior of other pathogenic viral nucleic acids in situ. Flow-based normalization, which incorporates measured daily flow rates entering treatment facilities and the population size of each sewershed catchment, is often suggested as the preferred normalization method for obtaining a per capita infection measure used for WBS.^10,30,31^ However, in systems with more pronounced I&I, flow rate trends may more closely align with precipitation and snowmelt patterns rather than changes in per capita contribution of fecal loading in-sewer.^10,13,24^ Additionally, the availability of high-resolution real-time flow monitoring data at both the subsewershed scale and at the WWPT influent is a challenge in some settings due to resource constraints, reliability of sensors due to harsh sewer environments and intermittent hydraulic conditions, and costs and labor associated with mounting and maintenance.^32^

To evaluate commonly used WBS normalization approaches over a range of site-specific and temporal levels of I&I, we collected extensive monitoring data over a 1 year period from a small, rural watershed. Monthly samples were collected at the WWTP influent and 11 additional sites experiencing various levels and types of I&I challenges. We evaluated trends for three viral pathogens in wastewater (SARS-CoV-2, norovirus GII, and rotavirus) and assessing several normalization scaling factors. Normalization scaling factors included three human fecal markers (HFMs): crAssphage, the hCYTB484 gene targeting the human mitochondrial genome (mtDNA), and the human-associated *Bacteroides* marker HF183. We also assessed normalization using physicochemical proxies for wastewater strength and human contribution to sewage: total suspended solids (TSS), chemical oxygen demand (COD), orthophosphate as phosphorus (PO_4_^3–^–P), and ammonia (NH_3_(aq)). Daily WWTP influent flow rate and population size were also used as normalization scaling factors. Our specific research objectives were to (1) evaluate associations between viral signals and TSS, COD, PO_4_^3–^–P, NH_3_(aq), crAssphage, HF183, and mtDNA, (2) examine how various normalization scaling factors affect observed site-specific and temporal variability of viral signals (with a focus on I&I and precipitation trends), and (3) assess whether normalization resulted in improved associations of wastewater data with clinical testing metrics relating to the incidence of disease caused by the monitored pathogens. Though some prior work has proposed normalization approaches that correct for sampling day wastewater compositions that differ from dry weather conditions,^10,13^ direct head-to-head comparison is needed to evaluate which approaches most effectively account for variability introduced by I&I-driven dilution and do not unintentionally introduce an additional layer of variability for relating biomarker signal measured in wastewater to per capita levels of pathogens in the community.

## Materials and Methods

The study was carried out on a sewer conveyance network serving a small town in southwest Virginia characterized by significant I&I (i.e., approximately 70–90% of WWTP influent flow). Before study initiation and following discussions with staff at the local utility and pilot testing, we preregistered our study protocols and a prespecified data management plan, available on the Open Science Framework.^33^ Given ethical and other concerns related to wastewater-based research for small populations^34^ and in line with our agreements with the local utility, we are not reporting the name or location of the study.

### Sampling Methods

1000 mL of wastewater samples were collected monthly (September 2022–August 2023) from the WWTP influent and 11 sewer nodes up-sewer from the WWTP, or from locations in the sewer conveyance network where wastewater flowed toward rather than away from the entrance to the WWTP. ISCO 6712 autosamplers (Teledyne ISCO, Lincoln, NE) were used to collect 24 h composite samples at four sites: (i) WWTP influent, (ii) down-sewer of a residential facility (∼1000 people) (Site S1), (iii) effluent from a series of septic tank effluent pumping (STEP) systems (Site S2), and (iv) a branch line up-sewer from a lift station (Site S3) **(** Figure 1). Two additional sampling events took place on July 18th and July 24th, 2023 at seven sites (WWTP influent, S1, S2, S9, S10, S11, and S12) to capture weekly resolution data for a month-long time span. Flow-weighted composite samples were collected at the WWTP influent and Site S1 using a 700 Area Flow Velocity Meter (Teledyne ISCO), and time-weighted composite samples were collected at all other composite sampling sites. Both composite sampling approaches targeted a collection of 48 samples collected over a 24 h period. Grab samples were collected at all other sewershed nodes on the morning of each sampling event (7–11 AM). Site characteristics (number of estimated individuals living up-sewer, etc.) and the total number of collection dates for each sampling site, including any deviations from the initial study design, are detailed in Table S1.

**Figure 1:**
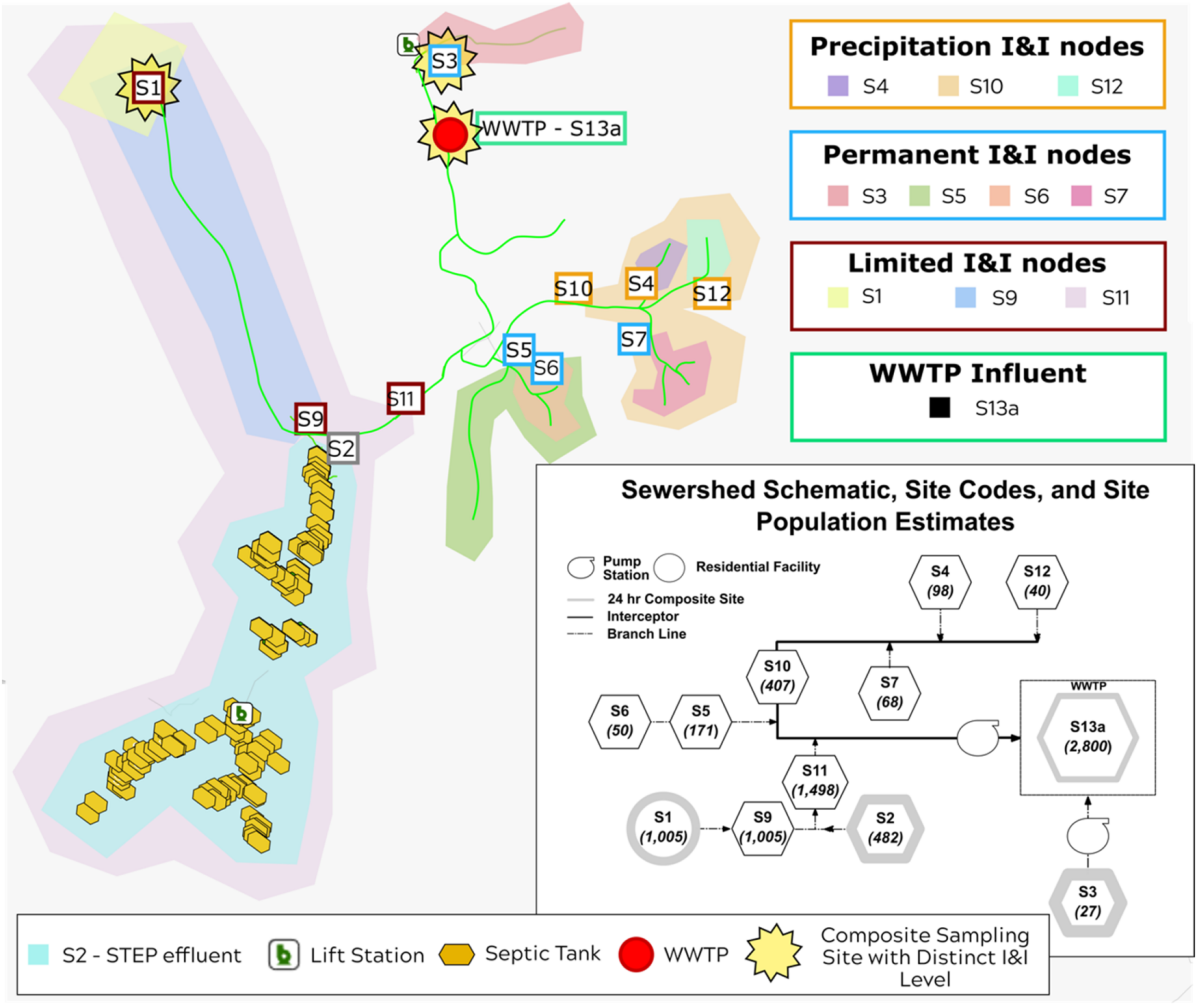
Deidentified sewershed map with demarcated nodes by I&I category, sampling approach, population residing up-sewer from the sewer collection node, and spatial relationship to other sewer nodes. In the sewershed schematic, the number of individuals residing up-sewer is indicated in parentheses. To avoid disclosing the specific location of the population served by the studied wastewater system due to ethical concerns related to the size of the residential population, we manually anonymized the map by shifting and scaling spatial coordinates while retaining the relative distances between sewer nodes and locations of nodes in reference to each other (i.e., S7, S4, and S12 were upstream of S10).

### Physicochemical, Precipitation, and the WWTP Influent Flow Rate Data

Dissolved oxygen, conductivity, temperature, and pH were measured on-site via a portable multiparameter meter (YSI Pro Plus, YSI, Yellow Springs, OH). Samples were transported to Virginia Tech on ice and remained at 4 °C until processing for COD (within 5 h of sample collection). Orthophosphate as phosphorus (PO_4_^3–^–P) and ammonia nitrogen as aqueous ammonia (NH_3_(aq)) were measured within 48 h of sample collection. TSS was measured within 72 h of sample collection following protocols specified in Table S2. Samples were acidified within 72 h after sample collection and then processed by ICP-MS for inorganic constituents (Table S2). Daily precipitation levels were recorded by wastewater plant staff using a Status Precision Rain Gauge placed at the WWTP. Daily wastewater flow rates were recorded by wastewater plant staff using a Teledyne ISCO 3010 Flow Transmitter by recording the total flow measurement at 8 AM and subtracting from the previous day’s 24 h total.

### Sample Concentration, Extraction, and ddPCR

Wastewater samples were spiked with MgCl_2_ to a final concentration of 25 mM upon arrival at the lab (on the day of collection) as described in Ahmed et al.^35^ 25–500 mL of wastewater, depending on the dilution of the sample, was vacuum-filtered onto 0.45 μm mixed cellulose ester filters in triplicate. The filters were ripped into 3 mm pieces using sterilized tweezers and placed in sterile 2 mL tubes. Though only one biological replicate was collected for each site and sampling date, each biological sample was filtered in triplicate to control for variability in filter processing. Samples were frozen at −80 °C prior to extraction, which was performed within 2 months after sample collection.

100 μL of total nucleic acid (TNA) was extracted from each filter replicate using the Zymo DNA/RNA Mini Prep Kit (Zymo Research, Irvine, CA) following the manufacturer’s protocol for TNA extraction, aliquoted into four aliquots of 25 μL, and stored at–80 °C prior to molecular analysis. Samples were processed in triplicate reactions using a droplet digital polymerase chain reaction (ddPCR) performed on a QX200 ddPCR System (Bio-Rad, Hercules, CA). All three filter replicates were processed for WWTP influent, and at least one filter replicate was processed for all viral targets. Three HFMs (crAssphage, HF183, and mtDNA) were quantified in at least one filter replicate for each site and sampling date. Thermal gradients were performed to determine the optimal annealing temperature for each assay.

All primers, probes, and standards used are reported in Table S3, alongside specific annealing temperatures and variations in cycling conditions for each target, as determined after optimization with thermal gradients. Except for the crAssphage and rotavirus probes, which were synthesized by Applied Biosystems (Foster City, CA), all primers and probes were prepared by IDT (Integrated DNA Technologies, Newark, New Jersey). Components and concentrations of each 20 μL reaction mixture are specified in Table S3. Triplicate NTC reactions were included for every 96-well plate to control for false positives. NTCs served as negative controls to rule out contamination introduced during the Mastermix and plate preparation. Triplicate reactions of standard (IDT gBlock or ATCC) at concentrations of ∼10^5^ and ∼10^3^ gene copies per mL were run for every 100 samples to control for false negatives. Reactions were scored as positive if the target was detected in at least three droplets, and at least 10,000 total droplets were generated. The fluorescence amplitude threshold distinguishing positive from negative droplets was set manually at the lower one-third of the space between the positive and negative droplet clusters to capture decreased fluorescence due to PCR inhibition.^36^ Extraction, ddPCR, and assay design specifications were designed to adhere to the guidelines from the Minimum Information for Publication of Quantitative Digital PCR Experiments as outlined in Table S4.^37^

### Spatial Analysis of the Sewershed

Whether I&I occurs primarily shortly after or during precipitation events or continuously as a result of hydrogeographic factors depends on site-specific characteristics. Consequently, the 11 up-sewer sampling sites were categorized into four distinct levels of I&I impacts based on average concentrations of COD as well as flow monitoring data from municipal and consulting engineers: “permanent I&I”, “precipitation-driven I&I”, “limited I&I”, and “WWTP influent” (Figure 1). WWTP influent was assigned as a fourth, separate &I level categories to encompass I&I impacts aggregated across the whole sewershed on normalized biomarker signal. This framework for demarcating I&I levels has been described previously^38^ and is detailed in Supporting Text 1.

Some analyses were limited to only three sites where composite samples were collected to account for potential variability introduced by grab sampling (Figure 1). These three sites also represented distinct I&I impact levels: permanent I&I (S3), limited I&I (S1), and WWTP influent (S13a), with average measured COD levels of 68 ± 82 mg/L, 474 ± 129 and 189 ± 153 mg/L, respectively (Table S5). These three sites were used to represent the impact of normalization on the biomarker signal at locations that reflect a range of dilution levels.

### Data Analysis

#### Calculation of Estimated Population of Sewershed

To serve as a relative proxy for flow contribution from each site to the WWTP and fecal loading for each site, we calculated static population estimates for each sewer node using household data from local utilities and occupancy estimates from Zillow and the Census Bureau’s recent population survey. Detailed methods are provided in Supporting Text 1.

#### Data Preparation for Normalization

Triplicate ddPCR reactions were averaged for each filter replicate processed. When multiple filter replicates were processed for one biological sample, we averaged the results across filter replicates. The total sample size for all ddPCR-derived biomarker and physicochemical data for each site and date is represented by the number of days where a sample was collected at each site, as specified in Table S1.

#### Data Preparation for Flow and Precipitation Data

A precipitation variable was derived based on cumulative precipitation summed across the 7 days prior to each sampling date (Figure S1). Detailed methods for tabulation of the precipitation variable are provided in Supporting Text 1.

#### Normalization Approaches

Concentrations of viral signal were normalized to physicochemical markers (e.g., virus concentration/TSS, virus concentration/COD) and HFMs (virus concentration/crAssphage concentration, virus concentration/mtDNA concentration, and virus concentration/HF183 concentration) following eq 1

1 $$C_{\mathrm{norm},X,i,j}=\frac{C_{i,j}}{X_{i,j}}$$

with *i* = sampling site in sewershed; *j* = sampling date; *C*_*i*,*j*_ = virus concentration log_10_(gene copies per mL); *X*_*i*,*j*_ = concentration of physicochemical or molecular marker of wastewater strength and population contribution to sewage; *C*_norm,*X*,*i*,*j*_ = normalized abundance = log_10_(gene copies per mL)/(units of physicochemical or molecular marker).

Flow-population normalization ((virus concentration × influent flow rate)/population served by entire sewershed) followed eq 2

2 $$C_{norm,Q,i,j}=C_{i,j}\times \frac{Q_{j}\times 1000\frac{mL}{L}\times 10^{6}\frac{gallons}{million\;gallons}\times 3.785\frac{liters}{gallon}}{P_{total}}$$

with *i* = sampling site in sewershed; *j* = sampling date; *C*_*norm*,*i*,*j*_ = normalized abundance log_10_(gene copies) per capita; *C*_*i*,*j*_ = virus concentration log_10_(gene copies per mL); *Q*_*j*_ = WWTP influent flow rate on sampling day (million gallons per day); *P*_total_ *=* total estimated population served by WWTP.

Virus concentrations were normalized to population data (virus concentration/number of individuals living up-sewer) following eq 3

3 $$C_{\mathrm{norm},P,i,j}=\frac{C_{i,j}}{P_{i}}$$

with *i* = sampling site in sewershed; *j* = sampling date; *C*_norm,*i*,*j*_ = normalized abundance log_10_(gene copies per mL) per capita; *C*_*i*,*j*_ = virus concentration log_10_(gene copies per mL); *P*_*i*_ *=* estimated number of individuals living up-sewer from sampling site.

The sample size for each normalization method by site and parameter used is provided in Table S6. To comparatively represent the disparate units in viral signal created across normalization measures, normalized concentrations of the viral signal are presented as normalized abundances. Units for normalized viral abundances with each metric are provided in Table S7.

#### Case Data Used in the Analysis

We obtained daily SARS-CoV-2 case data for the sewershed from the Virginia Department of Health. Because we lacked location-specific case data for norovirus, as a proxy we used weekly percentages of positive norovirus tests for the Southern census region of the US, as compiled by the CDC’s National Respiratory and Enteric Virus Surveillance System (NREVSS) (https://www.cdc.gov/surveillance/nrevss/). Similarly, for rotavirus, we used weekly NREVSS antigen detection estimates compiled for the US from September 2022 to August 2023.

To convert the weekly case data for norovirus and rotavirus into monthly aggregates (to better align with our monthly sampling), we computed a 4 week rolling average for NREVSS percent positive PCR or antigen tests. For daily COVID-19 case data, we computed a 28 day rolling average for the case counts to likewise convert the daily case data to monthly aggregates. Monthly aggregates for SARS-CoV-2, norovirus, and rotavirus cases were then correlated with monthly wastewater sampling results and then restricted to months for which we had collected wastewater samples (Table S1).

#### Statistical Analysis

The distributions of log_10_-transformed concentrations of molecular and physicochemical markers were checked for normality by using the Shapiro–Wilk test. Upon confirmation that ddPCR-derived biomarker concentrations and human input-based metrics were nonparametric, Kruskal–Wallis tests with post hoc Dunn tests, applied using a Bonferroni correction, were used to compare differences among the three composite sampling sites representing distinct levels of I&I impacts for each virus and normalization method. We set a significance threshold for post hoc Dunn tests at a p-value of 0.05. Coefficients of variation (CVs) were tabulated to determine the relative temporal variability of signal across dates for each site, normalization method, and assay by I&I categories. We used CV to evaluate variability, as opposed to variance, since the units in question were not otherwise directly comparable across normalization methods.

Spearman correlation tests were used to compare trends for wastewater virus signal vs proxies of wastewater strength measured in wastewater, unnormalized vs normalized viral trends, and wastewater-derived virus signal across normalization approaches vs case data, precipitation data, and the WWTP influent flow data. To assess the extent to which seasonal trends could potentially confound or otherwise impact differences in observed viral signals otherwise attributed to I&I under various normalization approaches, we replicated our correlation analysis using a detrending approach detailed in Supporting Text 1. Briefly, because our data set spanned only one full seasonal cycle (i.e., 12 months) and conventional methods for the decomposition and analysis of would-be seasonal trends require two or more full cycles (i.e., ≥24 months), we used a regression approach^39,40^ to detrend each variable separately and then used the residualized scores of the physicochemical and fecal marker variables after adjusting for month of the year for this additional Spearman correlation analysis. We set a threshold of significance for Spearman correlation tests at a *p*-value of 0.05.

All analyses were conducted using R (version 4.4.1), and primary statistical analyses were independently replicated. Figures were generated with R Studio (version 2024.04.2 + 764) using ggplot2, pheatmap, and ggstatsplot.

## Results and Discussion

### Wastewater Trends for Viruses and Wastewater Strength Proxies

Overall, we observed that ddPCR-derived HFMs mirrored the trends exhibited by norovirus GII and rotavirus at the WWTP influent, but the same was not true for the physicochemical markers (Figure 2). Conversely, we observed relatively stronger associations between physicochemical markers and the unnormalized SARS-CoV-2 signal at the WWTP influent compared to those observed for HFMs **(** Figure 2). Spearman correlation tests were significant between the crAssphage, mtDNA, and HF183 signals and the norovirus GII and rotavirus signals at WWTP influent (*p* < 0.05, *n* = 14), and correlation coefficients were all positive (Figure 2). Spearman correlations between trends for most physicochemical marker metrics and log_10_-transformed concentrations of SARS-CoV-2, norovirus GII, and rotavirus at WWTP influent were not statistically significant (Spearman’s correlation test; *p* < 0.05) (Figure 2). These observations suggest that normalization using HFMs could serve to account for variability introduced by I&I and other factors across sampling dates. After detrending the WWTP influent time series trends for unnormalized virus signals, HFMs, and physicochemical markers, positive and statistically significant associations were observed between SARS-CoV-2 signals and crAssphage, TSS, and COD and between norovirus GII signals and HF183 (Figure S2). These results indicate that seasonal effects may account for a meaningful portion of some of the observed associations, especially given the well-affirmed seasonal infection patterns in the US. during the time period studied for SARS-CoV-2 ^41^ as well as for noroviruses and rotaviruses.^42^ However, given that our time series did not span ≥24 months, we were not able to detrend our data using more conventional approaches, and with only one seasonal cycle of data, the overall import and significance of observed differences in correlations with and without our limited seasonal trend decomposition approach are unclear. To more extensively inform proper WBS data management practices, future work could re-evaluate the effect of various normalization approaches on data that span several full seasonal cycles.

**Figure 2:**
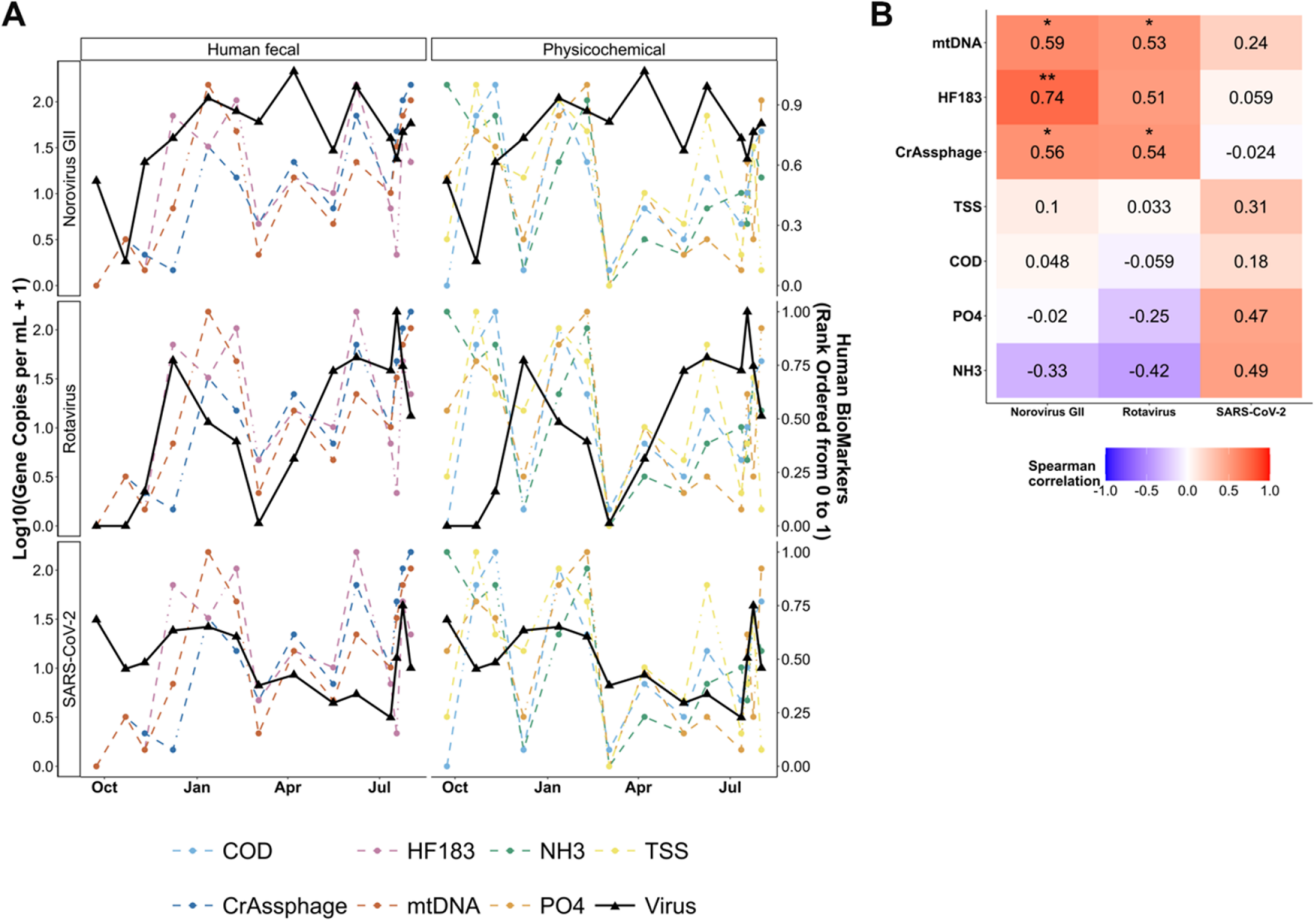
Unnormalized virus signals (SARS-CoV-2, norovirus GII, and rotavirus), human fecal marker signals (HF183, crAssphage, mtDNA), and physicochemical markers (PO_4_^3–^–P, TSS, COD, NH_3_(aq)) measured at wastewater influent with time. Panel (a) displays time series plots for human fecal biomarkers, physicochemical markers, and each virus. To facilitate a direct visual comparison of all seven human input-based normalizers alongside viral trends, we calculated the percentile rank for levels of each normalization metric across the study period from 0 to 1. Panel (b) displays a heat map with Spearman correlation coefficients between the viral signals and the candidate normalization scaling factors. Statistical significance is denoted as follows: *: *p* ≤ 0.05; **, *p* ≤ 0.01; and ***: *p* ≤ 0.001.

### Effects of Normalization across Site-Specific Levels of I&I

Between three of the sites used for comparison of I&I impacts (S1, S3, WWTP influent), differences in the baseline wastewater strength of the sewage were evident. Concentrations of all HFMs were highest at the up-sewer site with limited I&I impacts (S1), second highest at the WWTP influent (a site experiencing the strongest fecal loading in combination with I&I across the whole system), and lowest at the site with permanent I&I impacts (S3), representing <5% of the sewershed population (Figure S3). Thus, I&I appeared to dilute HFMs in the sewershed, as expected, although it is acknowledged that there could be other sources of variability in HFM signal measured at different sewershed sites due to distance from point sources of fecal shedding and resulting signal decay, variable proximity to point sources of inflow, and depth of the sewer pipe relative to the groundwater table.

To better contextualize how normalization approaches can alter associations between viral signals at varying levels of wastewater strength across sampling locations with differing levels of I&I, we compared unnormalized viral and HFM signals across sites with well-characterized I&I impact levels (S1, S3, WWTP influent). Overall, we observed indications that when the virus signal was normalized to physicochemical markers and population data, dilution-driven differences in virus signal were exacerbated, and normalized viral abundances were inflated at the permanent I&I sites, compared to the site with limited &I impacts. However, this was not the case for HFM-normalized data. When results were unnormalized or normalized to HFMs, viral concentrations of SARS-CoV-2 were not significantly different between sites (*p* > 0.10), and the site with permanent I&I impacts (S3) had the lowest median SARS-CoV-2 viral concentration (Figure 3). However, when normalized to physicochemical markers (COD, TSS, NH_3_(aq), PO_4_^3–^–P) and population data, the signal across sites was significantly different (*p* < 0.05) (Figure 3). Using these approaches, the permanent I&I sites also had a higher median normalized abundance of SARS-CoV-2 compared to WWTP influent and the site with limited I&I impacts (Figure 3), despite only representing <5% of the sewershed population (Figure 1). For rotavirus and norovirus GII, however, the highest median normalized abundance was observed at WWTP influent for all normalization techniques applied (Figure 3), though this was likely because norovirus GII and rotavirus detection up-sewer were less frequent than at WWTP influent (Figure S4). Because we lacked comprehensive, site-specific flow data, we were unable to incorporate flow when comparing site-specific differences in the viral signal.

**Figure 3:**
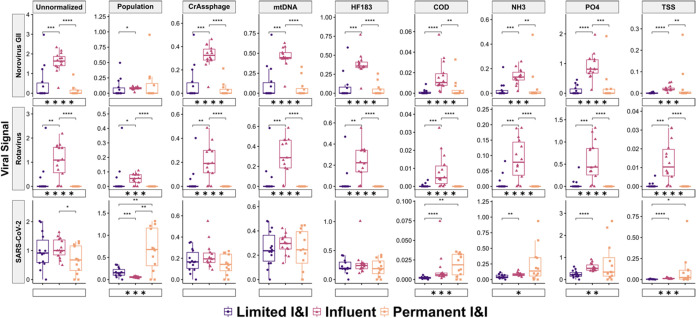
Effect of various normalization approaches on virus signals across three sites in distinct I&I categories. Box plots represent median unnormalized concentrations and normalized abundances across sampling dates. Unnormalized log_10_-transformed viral concentrations across sites are provided in units of log_10_(gene copies per mL + 1). However, since normalization resulted in different units for each normalization scaling factor, the term normalized abundance was used to encompass disparate units between normalization methods. Units for each normalization approach are detailed in Supporting Text 1. Significance levels for the Kruskal–Wallis tests are displayed under each plot using stars. Bracketed stars between I&I level sites represent *p*-value ranges for post hoc Dunn tests performed with an applied Bonferroni correction. Results are only shown for three sites where composite samplers were placed, which also represented distinct levels of I&I impacts (S1, S3, and WWTP influent). Significance levels are demarcated as follows: *: *p* < 0.05; **: *p* < 0.01; ***: *p* < 0.001; and **** *p* < 0.0001. Sample sizes for each site are denoted in Table S1 (S3: *n* = 12; S1: *n* = 14; S13a: *n* = 14).

Inflated signal for wastewater data scaled to physicochemical markers, where I&I were also most evident, may be due to the scale at which the physicochemical data were measured and interpreted. When measuring microorganisms and their nucleic acids, the focus is on detecting minute quantities of genetic material. Concentrations of DNA/RNA can vary widely even within small volumes of wastewater due to localized microbial activity, shedding rates, and the presence of specific pathogens. As a result of this microscale focus, concentrations of biomarkers in gene copies span several orders of magnitude in wastewater samples, whereas bulk measurements of physical or chemical properties may vary by, at most, 1 order of magnitude. COD, TSS, PO_4_^3–^–P, and NH_3_(aq) data are also often not log-transformed in analysis workflows, unlike quantitative molecular concentrations of pathogens and HFMs (e.g., crAssphage),^43^ which could in turn inadvertently introduce inconsistencies with scaling when comparing normalized viral signal across sites and over time.

Normalization to a HFM has been suggested as a scaling factor for viral wastewater signal to account for dilution impacts from I&I.^8,10,12,13^ In bench-scale work, viral concentrations have been found to directly decrease relative to HFMs such as crAssphage with increasing levels of dilution,^29^ though in situ evidence is needed to validate the suitability of crAssphage as a benchmark for determining accurate population-level infection trends relative to disparate shedding rates, decay rates, and in-sewer interactions between HFMs and the pathogen being scaled. Unlike normalization using physicochemical markers, normalization using HFMs is not limited to potential variability associated with scaling log-transformed viral RNA levels to untransformed physicochemical metrics. Though normalization using physicochemical-based sewage strength metrics appeared to substantively change the relative viral signal between site-specific levels of I&I, normalization with HFMs did not similarly alter the overall trends of SARS-CoV-2, norovirus GII, and rotavirus across the three composite sampling sites with distinct I&I impacts (S3, S1, and WWTP influent) (Figure 3). Additionally, if unnormalized concentrations of norovirus GII or rotavirus were significantly different between two sites with distinct I&I levels (limited I&I, permanent &I, and WWTP influent), they remained significantly different after normalization to crAssphage, mtDNA, and HF183 (Figure 3). We found exceptions, however, where viral concentrations of SARS-CoV-2, which were significantly different between WWTP influent and the site with permanent I&I impacts (S3) prior to normalization, were not significantly different after normalization to HF183, mtDNA, and crAssphage (Figure 3). Taken together, these data largely support the utility of HFMs to address site-specific differences in dilution levels, though more extensive mesocosm-based studies would be beneficial to confirm these effects.

The percent contribution of I&I to wastewater flow during dry weather periods is different across conveyance networks throughout the country^6,44^ and can exhibit as much as a 10-fold difference laterally along sewer conveyance lines.^45^ To account for these differences, normalization scaling factors should account for differences in wastewater strength while not unintentionally overinflating signal. Our findings illustrate how normalization using physicochemical marker parameters and population data has the potential to inadvertently inflate signal when I&I impacts are substantial and in so doing accentuate rather than diminish differences in the viral signal that are primarily due to dilution impacts.

### Effects of Normalization on Temporal Variability of Wastewater Signal by I&I Categories

Inflated virus signals for physicochemical marker-scaled data were observed not only for comparisons made across sites categorized by I&I levels but also as a function of precipitation levels over time. We observed that the temporal variability of the SARS-CoV-2 signal was highest for sites with permanent and precipitation-driven I&I impacts when normalized using NH_3_(aq), COD, and TSS data. It is noted that there was a relatively high infection prevalence of SARS-CoV-2 during the study period, with near 100% detection across all samples, and thus the variability we observed at precipitation I&I sites can more likely be attributed to the normalization approach used rather than detection variability. It was further noted that the median coefficient of variation (CV) across sampling dates for SARS-CoV-2 normalized abundance at each site were highest when normalized using physicochemical markers (PO_4_^3–^–P, COD, TSS, NH_3_(aq)) compared to unnormalized, flow-, and HFM-normalized data (Figure S5). When grouped by I&I categories, the median CV for all viruses when normalized to physicochemical markers was highest for sites categorized as having solely precipitation-driven I&I impacts (Figure S5).

Given literature-derived per capita loadings of TSS, COD, NH_3_(aq), and PO_4_^3–^–P,^46^ normalizing pathogen markers to these physicochemical measures has theoretical utility for estimating population normalized levels of viral nucleic acid from wastewater.^12,29^ However, in practice, human and industrial activity can alter concentrations of COD, TSS, NH_3_(aq), and PO_4_^3–^–P in sewer systems, and such impacts may not translate to commensurate alterations of pathogen signal in-sewer.^25^ COD, for instance, has a per capita estimated input to sewers of 135 g/day,^47^ yet significant reductions in COD concentrations have been observed during sewer transport that are unrelated to I&I.^22,48^ As a result, the use of bulk physical and chemical wastewater measurements to scale the virus signal may have heightened sensitivity to dilution from I&I. In turn, the temporal variability of virus signal normalized to such metrics may be elevated for sites where I&I levels are largely driven by precipitation events. These observed effects may not extend to other chemical analytes that have been proposed as normalization benchmarks and indicators of human urine/fecal load, such as sucralose or caffeine.^49^ However, unlike COD or TSS, such biomarkers do not reflect bulk organic matter or solid levels and are not routinely measured by WWTPs.

### Associations between Normalized Virus Signal and Precipitation Trends

Prior to any form of normalization, Spearman correlations between precipitation and SARS-CoV-2 (Spearman’s correlation test, ρ = -0.33, *p* > 0.10), norovirus GII (Spearman’s correlation test, ρ = 0.33, *p* > 0.10), and rotavirus (Spearman’s correlation test, ρ = 0.44, *p* > 0.10) concentrations at the WWTP influent were not significant. However, when normalized to some physicochemical markers like NH_3_(aq), Spearman correlations between precipitation patterns and SARS-CoV-2, norovirus GII, and rotavirus wastewater signal trends increased in correlational strength and significance (Figure 4). Correlations between virus concentrations and other physicochemical indicators (PO_4_^3–^–P and influent flow rate to the WWTP) show similar trends and significance (Figure 4). Associations between unnormalized and normalized temporal trends of the virus wastewater signal also decreased when the data were normalized using physicochemical markers (Figure S6). These observations suggest that using a physicochemical marker overly sensitive to wastewater dilution may cause wastewater virus signals to follow precipitation trends, amplifying bias rather than correcting for it. Although one of the primary reasons to adopt a normalization approach for WBS is to ensure wastewater pathogen data do not reflect dilution trends over time rather than infection trends, since the dilution of wastewater from I&I can alter the wastewater strength and consequently the viral signal, these data suggest that some normalization approaches could overinflate pathogen signals when wastewater is more diluted due to underlying differences in the scales for ddPCR-derived data compared to physicochemical data (e.g., log-transformed vs other). These data also align with our previously reported characterization of physicochemical signal and ddPCR-derived virus and HFM signal sensitivity to dry vs wet weather conditions,^38^ where bulk physicochemical parameters were significantly different (Mann–Whitney *U* test; *p* ≤ 0.05) on dry compared to wet weather days, whereas virus and HFM signal did not follow this trend. However, the extent to which sensitivity to dry and wet weather conditions may be heightened or diminished by the system size remains unclear. Scaling two metrics with different processing steps may also increase the variability due to sources of error unique to each processing workflow. Previous work^28^ suggests that NH_3_(aq) is a good candidate for normalizing wastewater pathogen signal in settings with pronounced dilution. However, NH_3_(aq)’s comparatively higher sensitivity to dilution compared to log_10_-transformed concentrations of viral and HFMs may result in exaggerated viral signals, especially after heavy precipitation events. Taken together, our findings suggest that normalization using NH_3_(aq), PO_4_^3–^–P, and other bulk physicochemical measures may overly account for changes in temporal patterns in the viral signal caused by dilution from I&I sources and therefore may inadvertently distort underlying infection trends.

**Figure 4:**
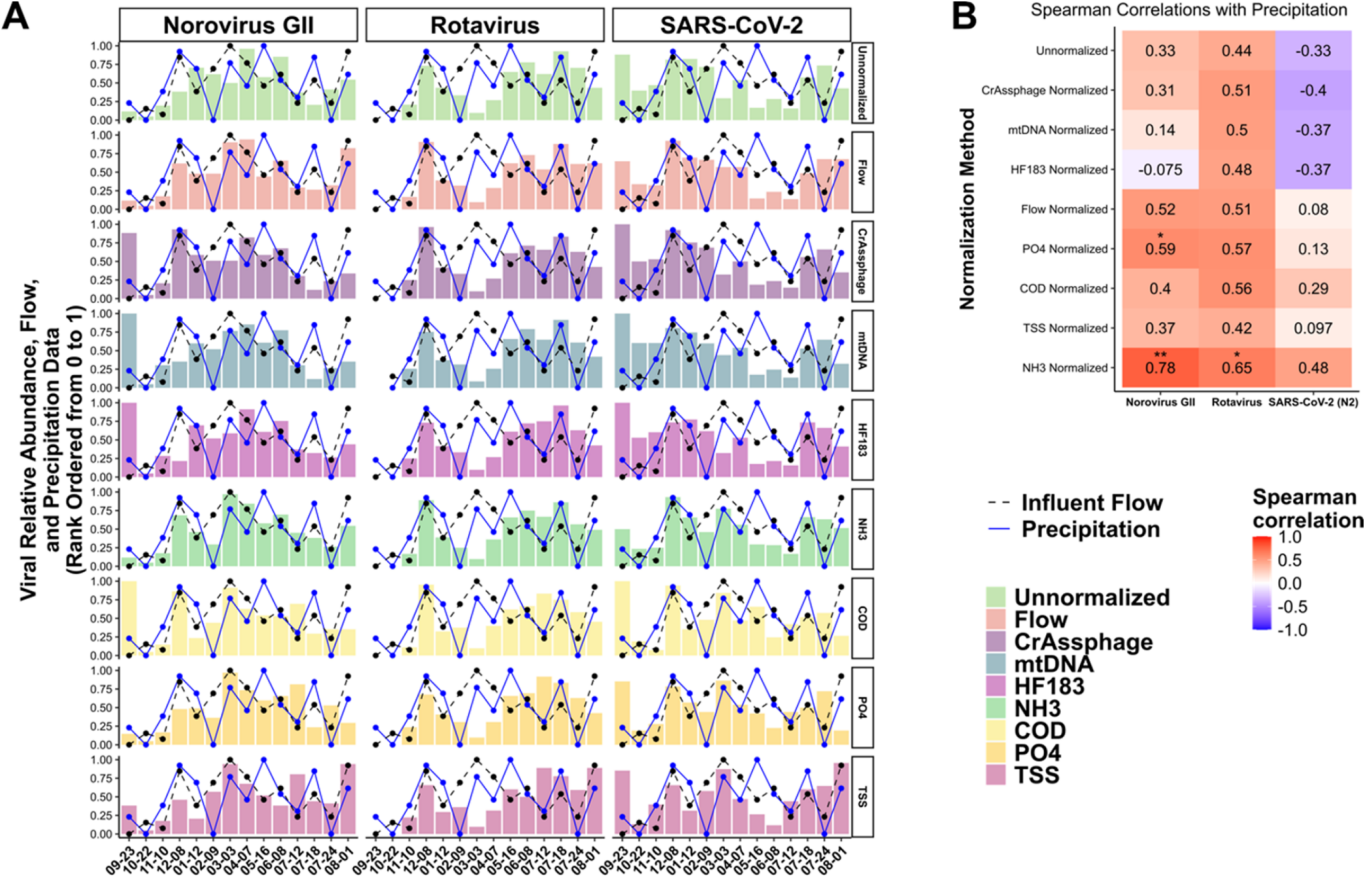
Spearman correlations between unnormalized and normalized virus wastewater trends and monthly precipitation levels. Panel (a) displays a time series viral signal by normalization method and virus. To allow direct visual comparison of all three viral targets across site-specific and temporal scales, we calculated their normalized abundance or unnormalized viral concentration for each site, viral target, and normalization method as the percentile rank of each sample’s unnormalized and normalized log-transformed ddPCR concentration on a scale from 0 to 1. Flow and precipitation data were also rank-ordered from 0 to 1 across sampling dates for visualization purposes. ddPCR concentrations obtained from samples filtered in triplicate were averaged for each site and date prior to analyses. For correlation analyses and plotting, only precipitation and influent flow rate data collected on the same day as wastewater data collection were used. Panel (b) displays heat maps depicting Spearman correlation values and associated significance levels between unranked, unnormalized concentrations, or normalized abundances for each virus and unranked precipitation data. Each row in panel (b) represents the viral signal for each normalization method. The Spearman correlation sample size for all viruses is 14. Statistical significance for Spearman correlation tests is denoted as follows: *: *p* ≤ 0.05; **, *p* ≤ 0.01; and ***: *p* ≤ 0.001.

In the heavily I&I impacted systems we studied, flow normalization resulted in stronger associations between precipitation and wastewater signal for SARS-CoV-2 (Spearman’s correlation test, ρ = 0.08, *p* > 0.10), norovirus GII (Spearman’s correlation test, ρ = 0.52, *p* ≤ 0.10), and rotavirus (Spearman’s correlation test, ρ = 0.51, *p* ≤ 0.10) (Figure 4), which was most likely the result of the influent flow being strongly correlated with precipitation (Figure S1). In most sewerage systems, the daily flow entering the WWTP is assumed to represent per capita wastewater contribution to the sewershed on the day of sampling^10^ and thus it is often used as a multiplication factor to improve estimates of viral wastewater concentrations per capita.^30,50^ Flow normalization has become one of the predominant normalization strategies used for CDC’s NWSS and other monitoring networks.^30^ It is important to recognize, though, that WWTP influent flow also reflects seasonal or precipitation-driven I&I patterns in-sewer in addition to flow from household sewage.^10,13,24^ Flow normalization may be necessary for sewersheds with extremely transient populations (i.e., college campuses, areas with tourism).^10^ However, heavily I&I impacted systems may be ill-suited for the use of flow-based normalization, since in such systems, the flow may often correlate more strongly with precipitation, snowmelt, and seasonal groundwater-level patterns than with fluctuations in the population contributing to the sewershed.^10,51^

### Impacts of Normalization Methods on Associations with Clinical Data

Generally, there has been disparate consensus on the effectiveness of normalization for improving or diminishing how well wastewater data correlate with clinical infection trend data.^12,26,52^ In many instances, normalization does not significantly improve and can even worsen the relationship between case data and wastewater trends.^26,53–55^

Associations between unnormalized wastewater signals for SARS-CoV-2 and COVID-19 case outcome data in our study were statistically significant (Spearman’s correlation test, *p* < 0.05) at the WWTP influent while not statistically significant at the up-sewer sites with permanent and limited I&I (Figure 5). However, unnormalized wastewater signals were better associated (Spearman’s correlation test, ρ = 0.54, *p* ≤ 0.05) at the WWTP influent compared to the up-sewer sites with permanent and limited I&I (Figure 5). Scaling the WWTP signal to physicochemical markers resulted in decreased associations between wastewater signal and case data (Spearman’s correlation test, ρ < 0.45, *p* > 0.10) at the WWTP influent (Figures 5 and S7). Scaling the WWTP influent signal to flow data and HF183 slightly decreased associations between wastewater signal and case data (Spearman’s correlation test, ρ < 0.52, *p* > 0.05) at the WWTP influent (Figures 5 and S7). For up-sewer sites, where there were limited associations between unnormalized wastewater signal and case data, we did not observe significant improvements after scaling using any approach, including HFMs. We also observed similar patterns for associations between case data and the normalized abundance of viral signal in wastewater across normalization methods for rotavirus and norovirus (Figure S7). However, these effects were observed using regional-scale case data and given the disparate scale between census-level case data and wastewater data from a community where <3,000 individuals reside; therefore, they likely do not meaningfully represent whether normalization allows for better approximation of community circulation of viruses from wastewater data. Norovirus and rotavirus circulation patterns, however, have had well-affirmed and consistently stable seasonality across the US. since 2021, which suggests that the studied community was not exempt from national-level circulation patterns.^42^

**Figure 5:**
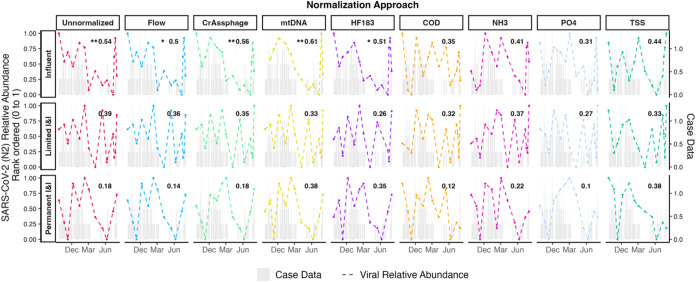
Relationship between SARS-CoV-2 normalized wastewater trends and the corresponding case data. In time series plots, to better visualize the effect of each normalization technique on trends for infection and wastewater virus levels, we rank-ordered viral signal from zero to one in each time series plot. The Spearman correlation coefficients and significance levels are denoted in the upper-right-hand corner of each subplot. Unranked log_10_-transformed viral concentrations and untransformed normalized abundances were used for all Spearman correlation analyses, as opposed to rank order scaling. Sample sizes for correlation analyses are listed in Table S8. Statistical significance for each Spearman correlation test is represented as follows: *: *p* ≤ 0.10; **: *p* ≤ 0.05; ***: *p* ≤ 0.01; and ****: *p* ≤ 0.001.

Most of the current literature to date on normalization approaches for wastewater monitoring focus explicitly on SARS-CoV-2 and span a period (2020–2022) when clinical testing coverage for SARS-CoV-2 was widespread with relatively high spatial and temporal resolution.^56^ Since a similar degree of granularity of clinical testing data is not typically available for many other nonreportable pathogens,^42,57^ using case data to assess the effectiveness of normalization as a means to reduce unwanted noise or biases in wastewater signals for other classes and types of pathogens remains challenging.

In our study, the only normalization approaches that maintained or slightly improved this association with the WWTP influent were those using HFMs (Figure 5). Specifically, case data were well-correlated with both unnormalized SARS-CoV-2 signal at WWTP influent (Spearman’s correlation test, ρ = 0.54, *p* ≤ 0.05) and that which was normalized to crAssphage and mtDNA (Spearman’s correlation test, ρ = 0.55, *p* ≤ 0.05) (Figure 5), which was also the case when exploring the use of 1 and 2 week lags (Figure S7). However, at the up-sewer sites with limited I&I and permanent I&I, normalizing sewer signal to HFMs did not statistically improve the association between case data and SARS-CoV-2 wastewater signal (Figure 5). These findings align with other studies showing the benefits of normalization to HFMs for wastewater data collected from the WWTP influent^24,58^ and suggest further that the use of HFMs may be particularly appropriate when normalizing sewer signal from sewersheds with substantial I&I impacts.

### Implications for Assessing Wastewater Surveillance Normalization Approaches in Larger Systems

Normalization via the use of scaling factors to adjust for variation in wastewater strength allows for the comparison of relative magnitudes of viral signal across different sewer networks, even in cases when baseline dilution levels from I&I at each sampling location are starkly different. The value to be gained from effective normalization is perhaps exemplified by the CDC’s National Wastewater Surveillance System (NWSS), which sources wastewater data from hundreds of sewer collection systems across the US.^59^ For systems with substantial I&I impacts, an effective normalization approach can help to reveal temporal patterns of viral shedding that might otherwise be overshadowed by precipitation-driven trends of sewage strength (i.e., level of dilution), while an ineffective normalization approach may inadvertently magnify the effect of precipitation on virus signal dilution.

In this study, we found that normalization using bulk physicochemical parameters (TSS, COD, PO_4_^3–^–P, and NH_3_(aq)) may inadvertently inflate rather than correct for site-specific and temporal dilution effects from I&I. As a result, biomarker signals normalized to bulk physicochemical parameters were more closely associated with precipitation trends rather than expected seasonal viral infection trends. In contrast, normalization using HFMs did not introduce similar distortions in relationships between viral wastewater signal and case data, precipitation data, and site-specific levels of I&I, confirming the utility of such markers as more reliable normalization metrics for future work. Observed misattribution using bulk physicochemical parameters would be expected to impact normalization in larger sewersheds as well. That said, for larger systems with known I&I hotspots that are considering using wastewater surveillance, results from our research indicate that it would be prudent to incorporate measures of sewage travel time (age) and localized I&I impacts to evaluate whether an adjusted normalization approach would be needed. However, further study is needed to assess the potential effects of I&I on normalization in larger systems.

### Optimizing Normalization Approaches for Smaller and Rural Systems

Delineating underlying pathogen–wastewater associations for sewersheds substantially impacted by I&I is especially important for many smaller rural systems, since they are, overall, less likely to effectively seek or receive many of the funding opportunities used by larger urban sewer systems to invest in proactive maintenance, line replacement, or sewer lining.^60^ Thus, in many rural settings, small, underfunded wastewater systems are at a higher risk of inadequate or postponed infrastructure maintenance and replacement, leading to increased I&I, and with it commensurate need for a suitable normalization approach that counteracts alterations of wastewater signal resulting from precipitation events.

Despite the potential benefits of WBS for rural areas, WBS has also not been as well-studied or as widely implemented in rural wastewater municipalities,^61^ which is problematic considering that most rural regions have comparatively less clinical surveillance data than their urban counterparts and are typically served by health districts with relatively fewer resources than urban areas.^62^ Given the potential benefits of WBS for augmenting clinical surveillance data and bolstering rural health departments^61^ upon implementation of additional ethical safeguards for sensitive health data collected from populations of this size,^61^ findings from this study can inform additional rural-focused WBS research and in turn expand the appropriate use of WBS in rural areas, particularly for those served by sewer systems with substantial I&I and sewage dilution impacts.

## Supplementary Material



## Notes

Text with additional experimental details, materials, and methods, including methods for demarcation of I&I impacts across the sewershed (Tables): checklist of reporting elements for publishing the ddPCR analysis of microbial targets; summary statistics for chemical oxygen demand by sampling site; sample sizes for normalized data at each site by normalization parameter; viral relative abundance units for normalization parameters; and sample sizes for Spearman correlation analyses (Figures); WWTP influent flow and precipitation; heat maps for detrended Spearman correlations between normalization scaling factors and virus signal; ddPCR-enumerated human fecal marker concentrations by I&I categories; frequency of detection percentages for viruses grouped by I&I categories; coefficients of variation for virus signal across sampling dates after normalization; heat maps for correlations between normalized and unnormalized wastewater virus signals; and comparisons between the virus signal and case data before and after normalization (PDF)
