# Supplementary material for: Comparative Assessment of Wastewater-Based Surveillance Normalization Methods to Improve Pathogen Monitoring in Rural Sewersheds
Source: Environ Sci Technol. 2025 May 27;59(22):11095–107. doi: 10.1021/acs.est.4c14485 (PMC12164260; doi:10.1021/acs.est.4c14485)
Supplement: Supplementary file 1 [file es4c14485_si_001.pdf]

SUPPORTING INFORMATION

Comparative Assessment of Wastewater Based Surveillance Normalization Methods to Improve Pathogen Monitoring in Rural Sewersheds

Amanda Darling,[a,b] Benjamin C. Davis,[a,c] Thomas Byrne,[d] Madeline Deck,[a] Gabriel E. Maldonado Rivera,[a] Sarah Price,[e] Amber Amaral-Torres,[a] Clayton Markham,[a] Raul A Gonzalez,[f] Peter J. Vikesland,[a] Leigh-Anne H. Krometis,[e] Amy Pruden,[a] and Alasdair Cohen[a,b*]

[a] Department of Civil and Environmental Engineering, Virginia Tech, Blacksburg, VA 24061, USA
[b] Department of Population Health Sciences, Virginia Tech, Blacksburg, VA 24061, USA
[c] Office of Research and Development, U.S. Environmental Protection Agency, Cincinnati, USA
[d] Genetics, Bioinformatics, and Computational Biology, Virginia Tech, Blacksburg, VA 24061, USA
[e] Department of Biological Systems Engineering, Virginia Tech, Blacksburg, VA 24061, USA
[f] Hampton Roads Sanitation District, Virginia Beach, VA 23455, USA

*Corresponding Author:
Alasdair Cohen
Department of Population Health Sciences
205 Duck Pond Dr.
Blacksburg, VA 24073, USA
E-mail address: alasdair.cohen@linacre.oxon.org (A.G. Cohen)

Supporting Information (SI) summary: 27 pages, 8 tables, 7 figures.

List of Supporting Information (SI) Text, Tables, and Figures

Supplemental Text 1: Details of Materials and Methods ***3***

Day-of Precipitation Impact Variable 3

Demarcation of I&I Impact Across Sewershed 4

Units for Each Wastewater Strength Proxy 5

Tables ***5***

Table S1. Sampling Site Characteristics 6

Table S2. Sample Processing Workflows 8

Table S3: ddPCR Single-plex Assays and Cycling conditions 9

Table S5: COD Levels at Each Site 16

Table S6: Sample Size for Normalized Data at Each Site by Parameter 17

Table S7: Viral Relative Abundance Units 18

Table S8: Sample Size for Spearman Correlation Analyses Between Viral Wastewater Signal and Case Data 19

Figures ***21***

Figure S1. Spearman correlation between precipitation and the WWTP influent flow rate 21

Figure S2. De-trended Spearman correlations between normalization scaling factors and virus signal in wastewater at the WWTP influent. Sample size is 14 for all Spearman correlation tests. Approach for removing seasonal trends from the data is described in Supplemental Text-1. 22

Figure S4. Frequency of detection for viruses grouped by I&I category. Septic tank effluent pumping system (STEP) denotes site S2. 24

Figure S5. Effect of normalization approaches on the coefficient of variation of virus signal across sampling dates for each sewershed monitoring location grouped by I&I level. Box plots represent median coefficient of variation across I&I levels. precipitation I&I (n = 3 sites); permanent I&I (n = 4 sites); limited I&I (n = 3 sites); the WWTP influent (n = 1 site). Sample size for the coefficient of variations calculation (n = 5-14 depending on the site and normalization method). Sample sizes are provided in Table S8. Coefficients of variation were calculated for relative abundances across dates at each site for each normalization approach and viral marker tested. The delineation of I&I impacts for each site are based on chemical oxygen demand levels on dry and wet weather days as described in the Supplemental Material. 25

Figure S6. Correlations between normalized and unnormalized wastewater virus signal at the WWTP influent 26

Figure S7. Relationship between normalized wastewater trends and corresponding case data. 27

Supplemental Text 1: Details of Materials and Methods

Day-of Precipitation Impact Variable

To assess the influence of day-of I&I impacts within the sewer system resulting from both immediate I&I and delayed infiltration on viral and antimicrobial resistance signal, we created a day-of precipitation-driven I&I variable. This variable accounted for the precipitation on the sampling day and the cumulative precipitation seven days preceding sample collection. This seven-day period was chosen based on observed correlations between influent flow rates and precipitation patterns. First, we calculated the Spearman correlation coefficients between daily WWTP flow rates and precipitation accumulated over various durations leading up to sampling, ranging from 0 to 8 days. We identified the highest significant correlation between cumulative precipitation and daily influent flow rate occurred for precipitation accumulated over seven days leading up to sampling. This sum was used to represent day-of precipitation impact to the sewer system for all analyses. A visualized correlation between cumulative precipitation and flow are provided in **Figure S1**.

Calculation of Population Estimates for Sewer Nodes

We used data provided by the local utility and wastewater treatment plant staff to estimate the number of households served across the entire sewer collection system, as well as the number of households up-sewer of each sample collection node. For residential addresses, to estimate the number of individuals residing in each household, we used publicly available data from Zillow (www.Zillow.com, Zillow Inc.). If the number of bedrooms was provided, we used this number as a crude estimate of the number of individuals residing at the address. If this data was not available, we used the average household size in the U.S. (2.5 people per household) in 2022 based on the Census Bureau's recent population survey recommendation to estimate the number of individuals residing at each address.

Detrending Approach Used for Spearman Correlation Tests

Given we did not collect more than one full seasonal cycle of data (i.e., >=24 months of monthly data), we could not perform seasonal trend decomposition using conventional locally estimated scatterplot smoothing (LOESS) or other similar techniques. To overcome this limitation, we detrended each variable included in our Spearman correlation analyses separately using the residualized scores of the physiochemical and fecal marker variables after adjusting for month of the year. Specifically, we first created a factor variable for the month of the year to account for seasonality. Then, we fitted a simple regression model for each variable against the month factor. The residuals from these regression models were used as de-trended values in our Spearman correlation tests.

Example: Detrending Approach for the Spearman Correlation Test between SARS-CoV-2 and HF183

**Variable Before Detrending**	**Sample size for Sampling Location (n)**	**Detrended Variable for Spearman Correlation**
Log10-transformed gene copies per mL of SARS-CoV-2	>12	Residuals from the regression between the month factor and Log10-transformed gene copies per mL of SARS-CoV-2
Log10-transformed gene copies per mL of HF183	>12	Residuals from the regression between the month factor and Log10-transformed gene copies per mL of HF183

Demarcation of I&I Impact Across Sewershed

We determined and validated the extent of Inflow and Infiltration (I&I) impacts from data concatenated from a flow monitoring campaign conducted by WWTP staff during periods with varying precipitation levels. Sites were identified as having moderate to heavy I&I impacts if sewer flow rates increased during or immediately after precipitation events compared to baseline dry weather flow. To distinguish between sites where I&I was permanent year-round or only present during or recently after precipitation events, we used average levels of chemical oxygen demand (COD) across dry and wet weather sampling events. Median strength wastewater averages 500 mg/L COD whereas 250 mg/L COD is regarded as weak wastewater according to industry standards (Metcalf and Eddy, 2003). Given this criterion for wastewater composition, we used 200 mg/L COD as a conservative cut-off for when sewage had evident I&I impacts during dry weather periods, since this cut-off was outside the range of typical wastewater. Sites with an average COD of less than 200 mg/L across dry weather sampling events were classified as sites with permanent inflow and infiltration. Sites with greater than 200 mg/L average COD on wet weather sampling events were classified as sites with limited I&I. Lastly, sites with significantly ($p<0.05$) different levels of COD on dry compared to wet weather periods were classified as sites with precipitation-driven I&I. Though I&I may be more pronounced directly or shortly after heavy precipitation events for all I&I-impacted sites, we distinguished between sites where I&I was present regardless of recent precipitation events to inform drivers of biomarker signal variability on dry compared to wet weather periods.

We also placed composite samplers at three sites with distinctly different I&I impacts (S3: Permanent I&I, S1: Limited I&I, and S13a: the WWTP influent) and average COD levels (S3: 73 mg/L, S1: 474 mg/L, and S13a: 202 mg/L) to directly compare observed dilution levels on biomarker signal.

I&I Category	Average COD on Dry Weather Days	Average COD on Wet Weather Days	COD on dry and wet weather days significantly different – p-value < 0.10 (Mann-Whitney U test)
**Permanent I&I**	<200 mg/L	NA	Yes or No
**Precipitation-driven I&I**	≥200 mg/L	-	Yes
**Limited I&I**	NA	>200 mg/L	No
**WWTP Influent**	NA	NA	NA

Units for Each Wastewater Strength Proxy

Wastewater Strength Proxy	Units
crAssphage	log_{10} (gene copies per mL)
mtDNA	log_{10} (gene copies per mL)
HF183	log_{10} (gene copies per mL)
PO_4^{3-}-P	mg/L
NH_3(aq)	mg/L
TSS	mg/L
COD	mg/L

Tables

Table S1. Sampling Site Characteristics

Site	Distance to Influent (ft)	Estimated Sewer Travel Time (h), Assuming min velocity of 2 ft^2/s	Number of Individuals Estimated to Live Up-Sewer	Watershed Catchment Size (km^2)	Elevation above sea level (ft)	I&I	Grab, Composite, Flow Composite, or Combination	Sampling Date and Method													
								9/23	10/22	11/10	12/8	1/12	2/9	3/3	4/7	5/16	6/8	7/12	7/18	7/24	8/1
1 (Residential Facility)	26,400	3.43	1,005	12.0	1655	Limited	Flow Composite	FC	G	FC	FC	C	C	C	C	FC	FC	C	C	C	C
2 (STEP effluent)	14,784	2.05	482	7.1	1554	Unknown	24-hr Composite	C	C	C	C	C	C	C	C	G	C	C	C	C	C
3	8,078	1.12	27	11.0	1587	Permanent	24-hr Composite	C	G	C	C	C	C	C	C	C	C	G	-	-	C
4	16,368	2.27	98	49.2	1596	Precipitation-Driven	Grab	G	-	G	-	G	G	G	G	G	G	-	-	-	-
5	10,084	1.40	171	28.6	1531	Permanent	Grab	G	G	G	G	G	G	-	-	-	G	G	-	-	G
6	10,618	1.47	50	28.6	1572.53	Permanent	Grab	G	G	G	G	G	G	G	G	G	G	G	-	-	G
7	16,262	2.26	68	45.4	1547	Permanent	Grab	G	G	G	G	G	G	G	G	G	G	G	-	-	-
8	3,485	0.48	127	-	1525.69	Limited	Grab	G	G	G	-	G	G	-	-	-	-	-	-	-	-
9	14,784	2.05	1,005	24.9	1554.69	Limited	Grab	G	G	G	G	G	G	G	G	G	G	G	G	G	G

10	13,147	1.83	407	95.5	1530	Permanent	Grab	G	G	G	G	G	G	-	G	G	G	G	G	G	G
11	12,989	1.80	1,498	33.6	1573.02	Limited	Grab	G	G	G	G	G	G	G	G	G	G	G	G	G	G
12	17,793	2.47	40	49.2	1618.14	Precipitation-Driven	Grab	G	G	G	G	G	G	G	G	G	G	G	G	G	G
13a-Influent	0	0	2,800	163.5	1552.47	-	Flow Composite	FC	FC	FC	FC	FC	G	G	FC	MC	C	MC	MC	FC	FC

FC: flow-weighted composite sample collected by sampling every instance the estimated average flow volume per half hour passed through the sewer
MC: morning composite sample collected from 6 – 11 AM. Sample collected every 5 minutes
C: 24-hour composite sample collected from ~6 – 8 AM the previous day to ~6 – 8 AM the morning of sampling. Samples collected every 30 minutes.
G: grab sample collected the morning of sampling from 6 – 11 AM
-: No sample collected

Table S2. Sample Processing Workflows

Measure	Units	Reference Method	Collection Bottle
**ICP-MS inorganics**	ppb or ppm	Standard Methods 3030D, 3125B[1]	Acid washed PP
**ddPCR targets**	Log10 (gene copies per mL + 1)	Minimum Information for Publication of Quantitative Digital PCR Experiments Guide (Abdel Nour & Pfaffl, 2022)	Autoclaved PP
**Chemical Oxygen Demand (COD)**	mg/L	Standard Methods 5220 D[1]	Autoclaved PP
**Total Suspended Solids (TSS)**	mg/L	*Standard Methods* 2540 D, E[1]	Autoclaved PP
**Ammonia-N (NH_3-N)**	mg/L	U.S. EPA Method 350.1[2]	Autoclaved PP
**Orthophosphate (PO_4^{3-})**	mg/L	U.S. EPA Method 365.3[3], following 40 CFR 136.6	Autoclaved PP

ICP-MS inorganics: Al, As, Ba, Cd, Cr, Co, Cl (ppm), Cu, Ca, Fe, Pb, Li, Mg, Mn, Mo, Ni, P, K, SO4 (ppm), Se, Si, Ag, Na, Sr, Sn, Ti, U, V, and Zn

PP: Polypropylene

ddPCR targets: Norovirus GII, SARS-CoV-2 (N2), Rotavirus, HF183, CrAssphage, mtDNA

1. APHA/AWWA/WEF. *Standard Methods for the Examination of Water and Wastewater*, 23rd ed.; American Public Health Association, American Water Works Association, and Water Environment Federation: Washington, DC, USA, 2018.
2. U.S. EPA. 1993. "Method 350.1: Nitrogen, Ammonia (Colorimetric, Automated Phenate)," Revision 2.0. Cincinnati, OH
3. U.S. Environmental Protection Agency (1978) EPA 365.3: Phosphorous, All Forms (Colorimetric, Ascorbic Acid, Two Reagent).

Table S3: ddPCR Single-plex Assays and Cycling conditions

Assay	F (5' - 3')	R (5' - 3')	Primer Manufacturer	Reaction Mixture	Probe	Probe Manufacturer	Standard	Cycling Conditions	PCR Supermix
Rotavirus	ACC ATC TWC ACR TRA CCC TCT ATG AG	GGT CAC ATA ACG CCC CTA TAG C	IDT	7 µL molecular grade H_2O<br>5 µL 1× Bio-Rad One-step RT-ddPCR Supermix for Probes<br>2 µL Reverse Transcriptase uL 300mM DTT<br>1 µL 20x Primer/Probe Stock (900 nM F/R primer; 250 nM of probe)<br>4 µL template nucleic acid	(FAM)-AGT TAA AAG CTA ACA CTG TCA AA-(MGB)	Applied Biosystems	ATCC #VR-2018-DQ	1. 50°C (60 minutes); 2. 95°C (10 minutes); 3. 40 cycles of: i) 95°C (30 seconds) ii) Annealing at 55°C (1 minute); 4. 98°C (10 minutes)	Biorad's One-Step RT-ddPCR Advanced Kit for Probes
CrAssphage	CAGAAGTACA AACTCCTAAAA AACGTAGAG	GATGACCAAT AAACAAGCCA TTAGC	IDT	6 µL molecular grade H_2O<br>10 µL 1× Bio-Rad ddPCR Supermix for Probes (No dUTP)<br>1 µL 20x Primer/Probe Stock (900 nM F/R primer; 250 nM of probe)<br>3 µL template nucleic acid	(FAM)-AGT TAA AAG CTA ACA CTG TCA AA-(MGB)	Applied Biosystems	TAATGCAGAA GTACAAACTCC TAAAAAACGT AGAGGTAGAG GTATTAATAAC GATTTACGTGA TGTAACTCGTA AAAAGTTTGAT GAACGTACTG ATTGTAATAAA GCTAATGGCTT GTTTATTGGTC ATCTTGAA	1. 95°C (10 minutes); 2. 40 cycles of: i) 94°C (30 seconds) ii) Annealing at 56°C (1 minute); 3. 98°C (10 minutes)	Biorad's ddPCR Supermix for Probes (No dUTP)
Norovirus GII	ATGTTCAGRTG GATGAGRTTCT CWGA	TCGACGCCATC TTCATTCACA	IDT	7 µL molecular grade H_2O<br>5 µL 1× Bio-Rad One-step RT-ddPCR Supermix for Probes<br>2 µL Reverse Transcriptase uL 300mM DTT	(HEX)-AGCACGTGGG AGGGCGATCG	IDT	AGAAGGTGGG ATGGACTTTTA CGTGCCAAGG CAGGAACCCA TGTTCAGGTGG ATGAGGTTTTC TGACTTGAGCA CGTGGGAGGG CGATCGCAATC TGGCTCCCAAT TTCGTGAATGA	1. 50°C (60 minutes); 2. 95°C (10 minutes); 3. 40 cycles of: i) 95°C (30 seconds) ii) Annealing at 56°C (1 minute); 4. 98°C (10 minutes)	Biorad's One-Step RT-ddPCR Advanced Kit for Probes

				1 µL 20x Primer/Probe Stock (900 nM F/R primer; 250 nM of probe)<br>4 µL template nucleic acid			AGATGGCGTC GAGTGACGCC AAC		
SARS-CoV-2 N2	TTA CAA ACA TTG GCC GCA AA	5’-GCG CGA CAT TCC GAA GAA-3’	IDT	7 µL molecular grade H_2O<br>5 µL 1× Bio-Rad One-step RT-ddPCR Supermix for Probes<br>2 µL Reverse Transcriptase uL 300mM DTT<br>1 µL 20x Primer/Probe Stock (900 nM F/R primer; 250 nM of probe)<br>4 µL template nucleic acid	(FAM)-ACAATTTGCCC CCAGCGCTTCA G-(BHQ1)	IDT	IDT 2019-nCoV_N_Positive Control	1. 50°C (60 minutes); 2. 95°C (10 minutes); 3. 40 cycles of: i) 95°C (30 seconds) ii) Annealing at 55°C (1 minute); 4. 98°C (10 minutes)	Biorad’s One-Step RT-ddPCR Advanced Kit for Probes
HF183	ATCATGAGTTC ACATGTCCG	CGTAGGAGTTT GGACCGTGT	IDT	6 µL molecular grade H_2O<br>10 µL 1× Bio-Rad ddPCR Supermix for Probes (No dUTP)<br>1 µL 20x Primer/Probe Stock (900 nM F/R primer; 250 nM of probe)<br>3 µL template nucleic acid	(FAM)-AGT TAA AAG CTA ACA CTG TCA AA-(MGB)	IDT	TAGTAATGAAT CATGAGTTCAC ATGTCCGCATG ATTAAAGGTAT TTTCCGGTAGA CGATGGGGAT GCGTTCCATTA GATAGTAGGC GGGGTAACGG CCCACCTAGTC AACGATGGAT AGGGGTTCTGA GAGGAAGGTC CCCCACATTGG AACTGAGACA CGGTCCAAACT CCTACGGGAG G	1. 95°C (10 minutes); 2. 40 cycles of: i) 94°C (30 seconds) ii) Annealing at 58°C (1 minute); 3. 98°C (10 minutes)	Biorad’s ddPCR Supermix for Probes (No dUTP)

mtDNA	CAATGAATCTG AGGAGGCTAC	CGTGCAAGAA TAGGAGGTG	IDT	6 µL molecular grade H_2O 10 µL 1× Bio-Rad ddPCR Supermix for Probes (No dUTP) 1 µL 20x Primer/Probe Stock (900 nM F/R primer; 250 nM of probe) 3 µL template nucleic acid	(FAM)-AGT TAA AAG CTA ACA CTG TCA AA-(MGB)	IDT	TAGTAATGACA ATGAATCTGAG GAGGCTACTCA GTAGACAGTCC CACCCTCACAC GATTCTTTACC TTTCACTTCAT CTTACCCTTCA TTATTGCAGCC CTAGCAGCACT CCACCTCCTAT TCTTGCACGTA GTAATGA	1. 95°C (10 minutes); 2. 40 cycles of: i) 94°C (30 seconds) ii) Annealing at 59°C (1 minute); 3. 98°C (10 minutes)	Biorad's ddPCR Supermix for Probes (No dUTP)

Table S4: dMIQE2020 checklist for authors, reviewers and editors.

ITEM TO CHECK	PROVIDED	COMMENT
**1. SPECIMEN**		
Detailed description of specimen type and numbers	**N**	N/A
Sampling procedure (including time to storage)	**Y**	•Sampling procedures detailed in Sampling Methods section of Materials & Methods<br>•Storage temperature between sampling and concentration: 4 C for <6 hrs<br>•Storage temperature of concentrated filters before extraction: -80 C
Sample aliquotation, storage conditions and duration	**Y**	•TNA aliquoted into aliquots of 25 uL and kept in -80 C before processing
**2. NUCLEIC ACID EXTRACTION**		
Description of extraction method including amount of sample processed	**Y**	•100 mL of total nucleic acid (TNA) was extracted from each filter replicate using the Zymo DNA/RNA Mini Prep Kit (Zymo Research, Irvine, CA) following the manufacturer's protocol for TNA extraction, aliquoted into four aliqouts of 25 mL, and stored at -80°C prior to molecular analysis
Volume of solvent used to elute/resuspend extract	**N**	
Number of extraction replicates	**Y**	•One extraction per filter processed<br>•Three filters processed per biological sample
Extraction blanks included?	**Y**	•Extraction blanks were processed for each TNA extraction<br>•We did not include extraction blanks for the majority of dPCR runs due to costs associated with ddPCR<br>•ddPCR runs where extraction blanks were included were not positivee detect for any assay<br>•We did not include extraction blanks as part of our analysis
**3. NUCLEIC ACID ASSESSMENT AND STORAGE**		
Method to evaluate quality of nucleic acids	**N**	
Method to evaluate quantity of nucleic acids (including molecular weight and calculations when using mass)	**Y**	•We enumerated concentrations of double stranded DNA in ng/uL in each sample extract using Qubit<br>• We did not include DNA concentrations in our formal analysis
Storage conditions: temperature, concentration, duration, buffer, aliquots	**Y**	•Storage temperature of TNA extracts before dPCR: -80 C

Clear description of dilution steps used to prepare working DNA solution	**Y**	•Assays we performed 1:10 dilutions of sample extract for: crAssphage, HF183, mtDNA<br>•Dilutions were performed using DNAse/RNAse-free water
**4. NUCLEIC ACID MODIFICATION**		
Template modification (digestion, sonication, pre-amplification, bisulphite etc.)	**N**	N/A
Details of repurification following modification if performed	**N**	N/A
**5. REVERSE TRANSCRIPTION**		
cDNA priming method and concentration	**N**	N/A
One or two step protocol (include reaction details for two step)	**Y**	•One-step protocol used for QX200 ddPCR system
Amount of RNA added per reaction	**Y**	•Detailed in Table S3
Detailed reaction components and conditions	**Y**	•Detailed in Table S3
Estimated copies measured with and without addition of RT*	**N**	N/A
Manufacturer of reagents used with catalogue and lot numbers	**Y**	•Detailed in Table S3
Storage of cDNA: temperature, concentration, duration, buffer and aliquots	**N**	N/A
**6. dPCR OLIGONUCLEOTIDES DESIGN AND TARGET INFORMATION**		
Sequence accession number or official gene symbol	**Y**	•Detailed in Table S3
Method (software) used for design and *in silico* verification	**Y**	•We used Geneious prime software to perform *in silico* validation of primer and probe sequences, optimize primer specificity and performance for ddPCR, and reduce primer dimer & false positives
Location of amplicon	N	
Amplicon length	**Y**	•Detailed in Table S3
Primer and probe sequences (or amplicon context sequence)**	**Y**	•Detailed in Table S3
Location and identity of any modifications	**N**	N/A
Manufacturer of oligonucleotides	**Y**	•Detailed in Table S3
**7. dPCR PROTOCOL**		

Manufacturer of dPCR instrument and instrument model	**Y**	•Detailed in Sample, Concentration, Extraction, and ddPCR section of materials & methods<br>•QX200 ddPCR System (Bio-Rad, Hercules, CA)
Buffer/kit manufacturer with catalogue and lot number	**Y**	•Detailed in Table S3
Primer and probe concentration	**Y**	•Detailed in Table S3
Pre-reaction volume and composition (incl. amount of template and if restriction enzyme added)	**Y**	•Detailed in Table S3
Template treatment (initial heating or chemical denaturation)	**N**	N/A
Polymerase identity and concentration, Mg++ and dNTP concentrations***	**Y**	•Detailed in Table S3
Complete thermocycling parameters	**Y**	•Detailed in Table S3
**8. ASSAY VALIDATION**		
Details of optimisation performed	**Y**	•Thermal gradients were performed prior to running samples for each assay to determine the optimal annealing temperature<br>•Dilution series were performed to optimize assay performance (e.g., optimal separation of positive and negative flourescent amplitude if inhibition was present, optimal dilution to avoid samples surpassing ddPCR upper limit of quantification)
Analytical specificity (vs. related sequences) and limit of blank (LOB)	**N**	•Given the inherent advantages of our chosen quantification method, which offers higher sensitivity, specificity, and precision for detecting true positives compared to qPCR, we did not include a formal limit of blank (LOB) in this study.<br>•Our QA/QC methodology - triplicate ddPCR reactions for each sample, requiring a minimum of 3 positive droplets to confirm a true positive, and generating >10,000 droplets per sample to ensure robust detection - provides sufficient rigor for reliable quantification. Additionally, determining and reporting LOBs were outside the primary scope and objectives of this study.
**Analytical** sensitivity/LoD and how this was evaluated +A65[@Column1]	**N**	•Given the inherent advantages of our chosen quantification method, which offers higher sensitivity, specificity, and precision for detecting true positives compared to qPCR, we did not include a formal limit of detection (LOD) in this study.<br>•Our QA/QC methodology - triplicate ddPCR reactions for each sample, requiring a minimum of 3 positive droplets to confirm a true positive, and generating >10,000 droplets per sample to ensure robust detection - provides sufficient rigor for reliable quantification. Additionally, determining and reporting LODs were outside the primary scope and objectives of this study.
Testing for inhibitors (from biological matrix/extraction)	**N**	•We did not test for inhibitors specifically but modified our thresholding protocol to include droplets in the lower one third between the negative and positive droplet line to account for potential inhibition<br>•We also optimized dilution patterns

9. DATA ANALYSIS		
Description of dPCR experimental design	**Y**	
Comprehensive details negative and positive of controls (whether applied for QC or for estimation of error)	**Y**	•Detailed in materials & methods
Partition classification method (thresholding)	**Y**	•Detailed in materials & methods
Examples of positive and negative experimental results (including fluorescence plots in supplemental material)	**N**	N/A
Description of technical replication	**Y**	•We processed sample extracts in technical triplicate ddPCR reactions
Repeatability (intra-experiment variation)	**Y**	
Reproducibility (inter-experiment/user/lab etc. variation)	**Y**	
Number of partitions measured (average and standard deviation)	**Y**	•We only included ddPCR reactions in our final analysis if amplification created at least 10,000 droplets
Partition volume	**N**	~2 nL per droplet as per Bio-Rad's specifications
Copies per partition (λ or equivalent) (average and standard deviation)	**N**	
dPCR analysis program (source, version)	**Y**	QX Manager Software, Standard Edition v2.0
Description of normalisation method	**Y**	•Detailed in materials & methods
Statistical methods used for analysis	**Y**	•Detailed in materials & methods
Data transparency	raw data available on request:	

Table S5: COD Levels at Each Site

Site	COD (mg/L)				I&I Level
	Mean	Standard deviation	Minimum	Maximum	
S1	474	129	308	678	Limited I&I
S2	348	301	140	1307	STEP
S3	68	82	6	311	Permanent I&I
S4	515	371	17	1110	Precipitation I&I
S5	121	98	7	315	Permanent I&I
S6	113	101	20	344	Permanent I&I
S7	56	28	19	114	Permanent I&I
S9	447	185	20	759	Limited I&I
S10	231	299	12	1127	Precipitation I&I
S11	416	200	20	720	Limited I&I
S12	499	354	31	964	Precipitation I&I
S13a	189	153	20	631	Influent

Table S6: Sample Size for Normalized Data at Each Site by Parameter

Site	I&I Level	Sample Size after Normalization for all Viruses (N)							
		COD	crAssphage	HF183	Influent Flow	mtDNA	NH_3(aq)	PO_4^{3-}-P	TSS
S13a	Influent	14	14	14	14	14	14	14	14
S1	Limited I&I	14	14	14	14	14	14	14	14
S11	Limited I&I	11	11	11	11	11	10	11	11
S9	Limited I&I	11	11	11	11	11	11	11	11
S3	Permanent I&I	12	12	12	12	12	12	12	12
S5	Permanent I&I	6	6	6	6	6	6	6	5
S6	Permanent I&I	9	9	9	9	9	9	9	9
S7	Permanent I&I	8	8	8	8	8	8	8	8
S10	Precipitation I&I	10	10	10	10	10	9	10	10
S12	Precipitation I&I	11	11	11	11	11	10	11	11
S4	Precipitation I&I	6	6	6	6	6	6	6	6
S2	STEP effluent	14	14	14	14	14	13	14	14

Table S7: Viral Relative Abundance Units

Normalization Metric	Relative Abundance Units
Influent Flow	Virus $\log_{10}$ (gene copies / capita)
crAssphage	Virus $\log_{10}$ (gene copies per mL +1) / crAssphage $\log_{10}$ (gene copies per mL +1)
mtDNA	Virus $\log_{10}$ (gene copies per mL +1) / mtDNA $\log_{10}$ (gene copies per mL +1)
HF183	Virus $\log_{10}$ (gene copies per mL +1) / HF183 $\log_{10}$ (gene copies per mL +1)
PO_4^{3-}-P	Virus $\log_{10}$ (gene copies per mL +1) / PO_4^{3-}-P mg/L
NH_3(aq)	Virus $\log_{10}$ (gene copies per mL +1) / NH_3(aq) mg/L
TSS	Virus $\log_{10}$ (gene copies per mL +1) / TSS mg/L
COD	Virus $\log_{10}$ (gene copies per mL +1) / COD mg/L

Table S8: Sample Size for Spearman Correlation Analyses Between Viral Wastewater Signal and Case Data

Site	Virus	Normalization Metric	Lag (days)	N
Influent	Norovirus GII	HF183	14	14
			7	14
			0	14
		mtDNA	14	14
			7	14
			0	14
		CrAssphage	14	14
			7	14
			0	14
		PO_4^{3-}-P	14	14
			7	14
			0	14
		NH_3(aq)	14	14
			7	14
			0	14
		TSS	14	14
			7	14
			0	14
		COD	14	14
			7	14
			0	14
		Flow-Population	14	14
			7	14
			0	14
		Unnormalized	14	14
			7	14
			0	14
	SARS-CoV-2 (N2)	HF183	14	13
			7	12
			0	11
		mtDNA	14	13
			7	12
			0	11
		CrAssphage	14	13
			7	12
			0	11
		PO_4^{3-}-P	14	13
			7	12
			0	11
		NH_3(aq)	14	13
			7	12
			0	11
		TSS	14	13
			7	12
			0	11
		COD	14	13
			7	12

Site	Virus	Normalization Metric	Lag (days)	N
			0	11
		Flow-Population	14	13
			7	12
			0	11
		Unnormalized	14	13
			7	12
			0	11
	Rotavirus	HF183	14	14
			7	14
			0	14
		mtDNA	14	14
			7	14
			0	14
		CrAssphage	14	14
			7	14
			0	14
		PO_4^{3-}-P	14	14
			7	14
			0	14
		NH_3(aq)	14	14
			7	14
			0	14
		TSS	14	14
			7	14
			0	14
		COD	14	14
			7	14
			0	14
		Flow-Population	14	14
			7	14
			0	14
		Unnormalized	14	14
			7	14
			0	14

Figures

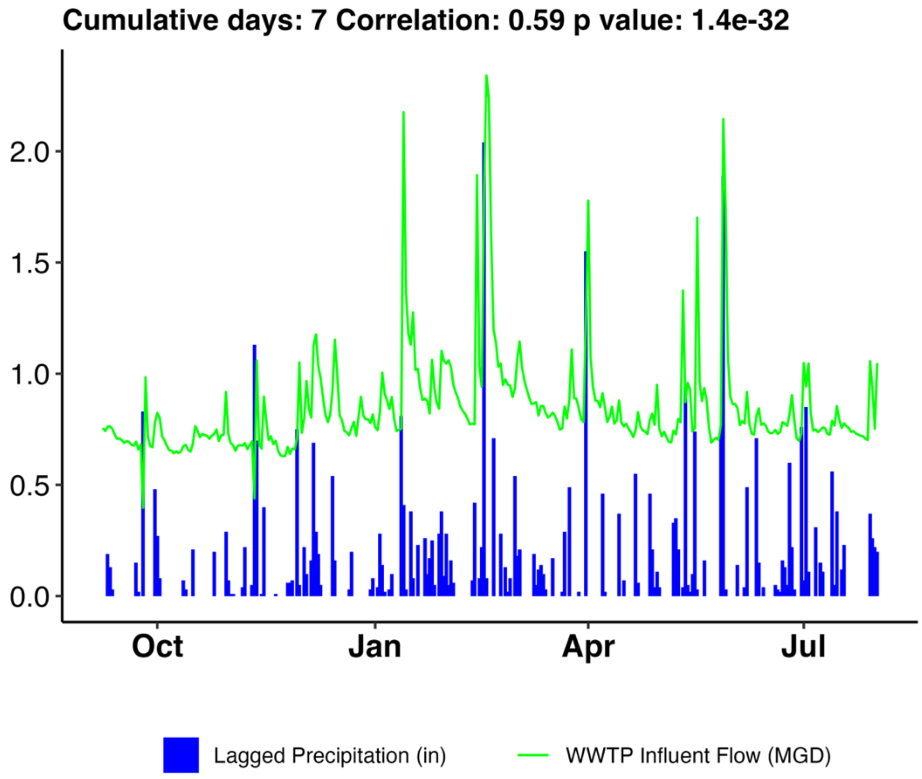

Figure S1. Spearman correlation between precipitation and the WWTP influent flow rate

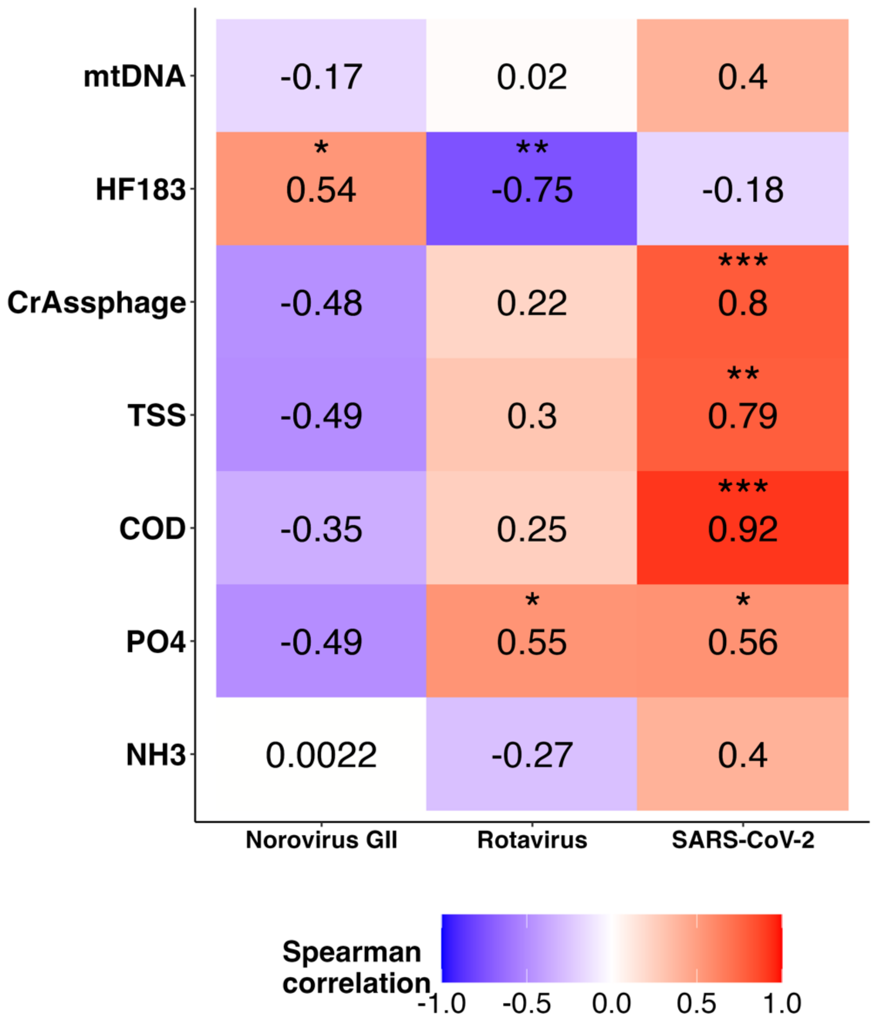

Figure S2. De-trended Spearman correlations between normalization scaling factors and virus signal in wastewater at the WWTP influent. Sample size is 14 for all Spearman correlation tests. Approach for removing seasonal trends from the data is described in Supplemental Text-1.

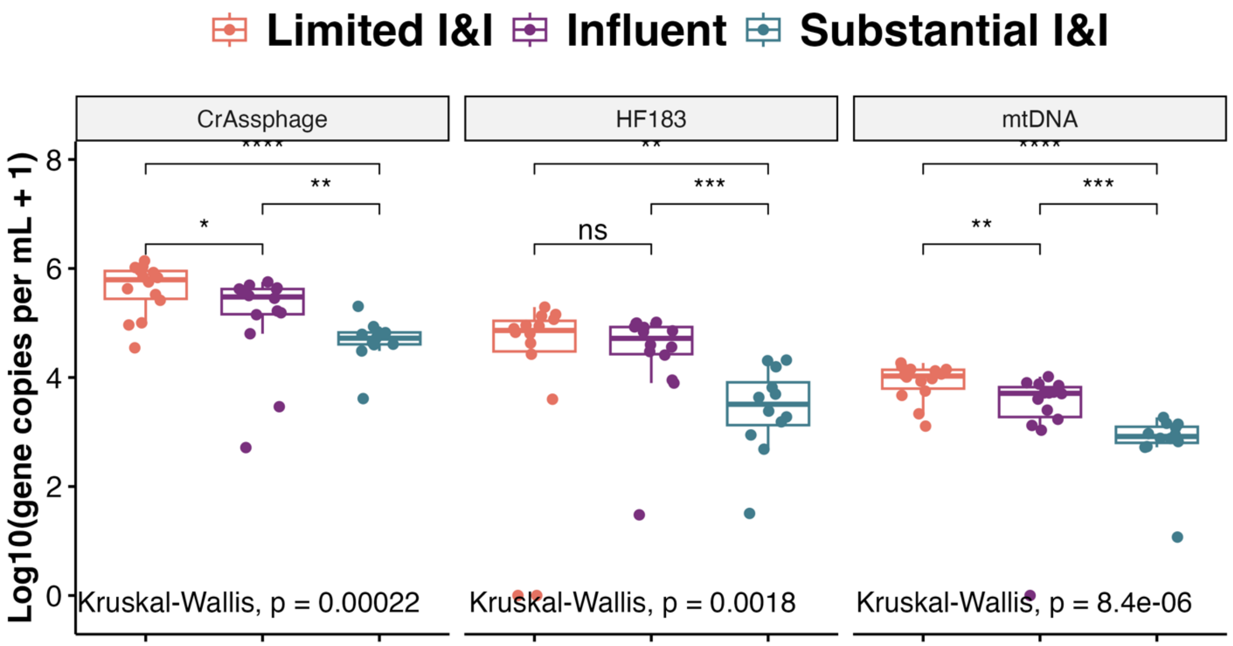

Figure S3. Concentrations of human fecal markers by I&I level**.** Log_{10} transformed concentrations of gene copies per mL given for each human excreta biomarker at three of the sewershed sites sampled with distinct levels of I&I impact (S1-Limited I&I, S3-Permanent I&I, and S13a-the WWTP influent) are shown. Significance levels are demarcated as follows: ns: p> 0.05; *: p≤0.05; **: p≤0.01; ***: p≤0.001.

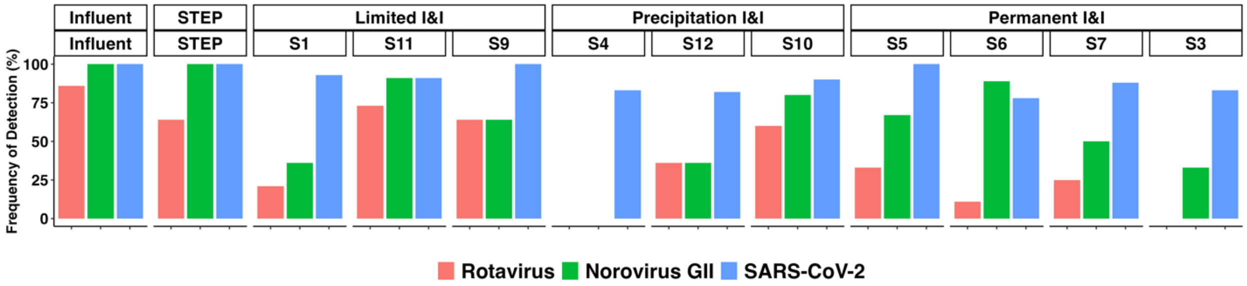

Figure S4. Frequency of detection for viruses grouped by I&I category. Septic tank effluent pumping system (STEP) denotes site S2.

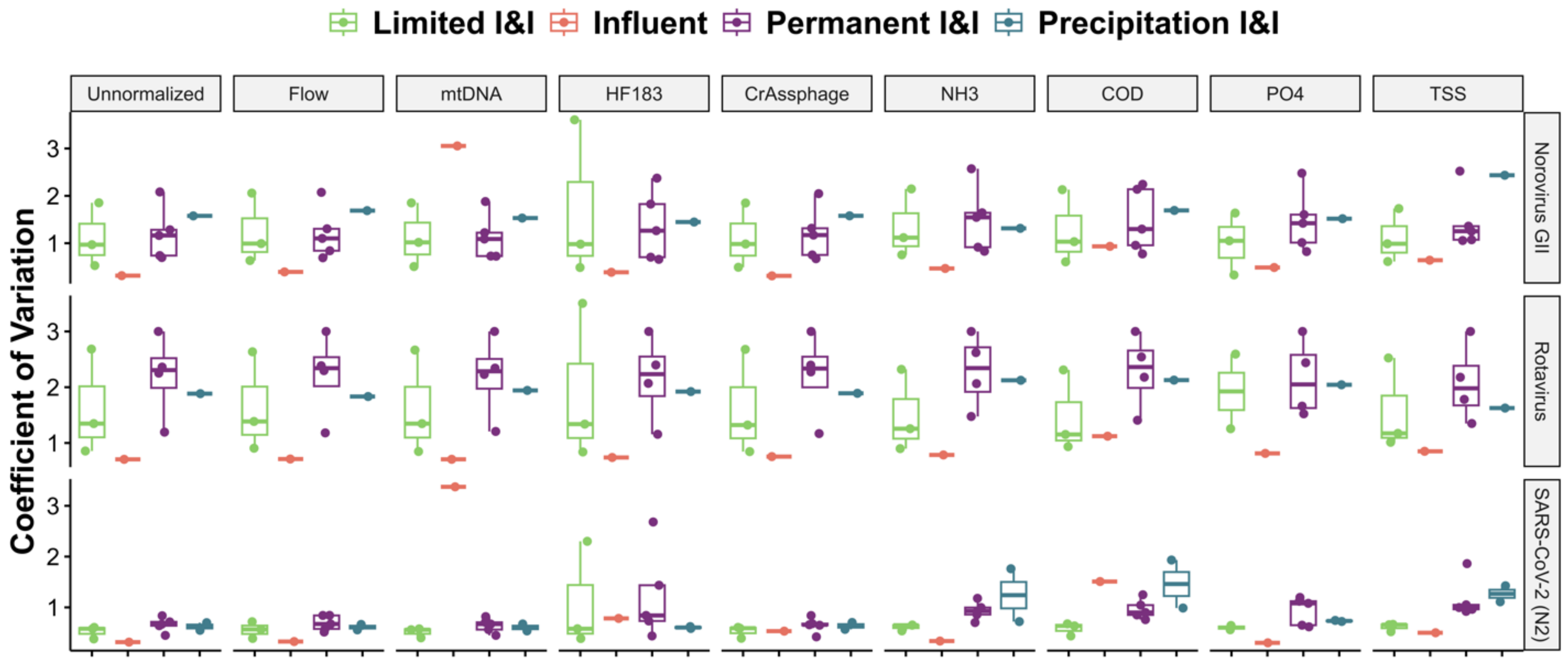

Figure S5. Effect of normalization approaches on the coefficient of variation of virus signal across sampling dates for each sewershed monitoring location grouped by I&I level. Box plots represent median coefficient of variation across I&I levels. precipitation I&I (n = 3 sites); permanent I&I (n = 4 sites); limited I&I (n = 3 sites); the WWTP influent (n = 1 site). Sample size for the coefficient of variations calculation (n = 5-14 depending on the site and normalization method). Sample sizes are provided in Table S8. Coefficients of variation were calculated for relative abundances across dates at each site for each normalization approach and viral marker tested. The delineation of I&I impacts for each site are based on chemical oxygen demand levels on dry and wet weather days as described in the Supplemental Material.

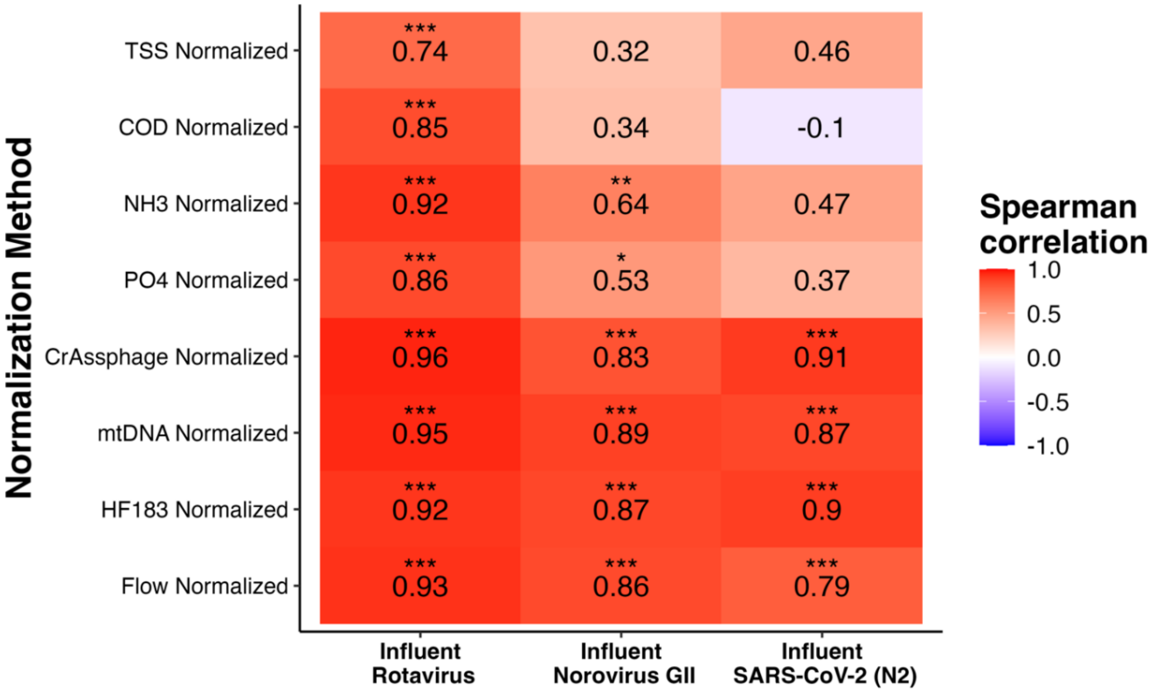

Figure S6. Correlations between normalized and unnormalized wastewater virus signal at the WWTP influent

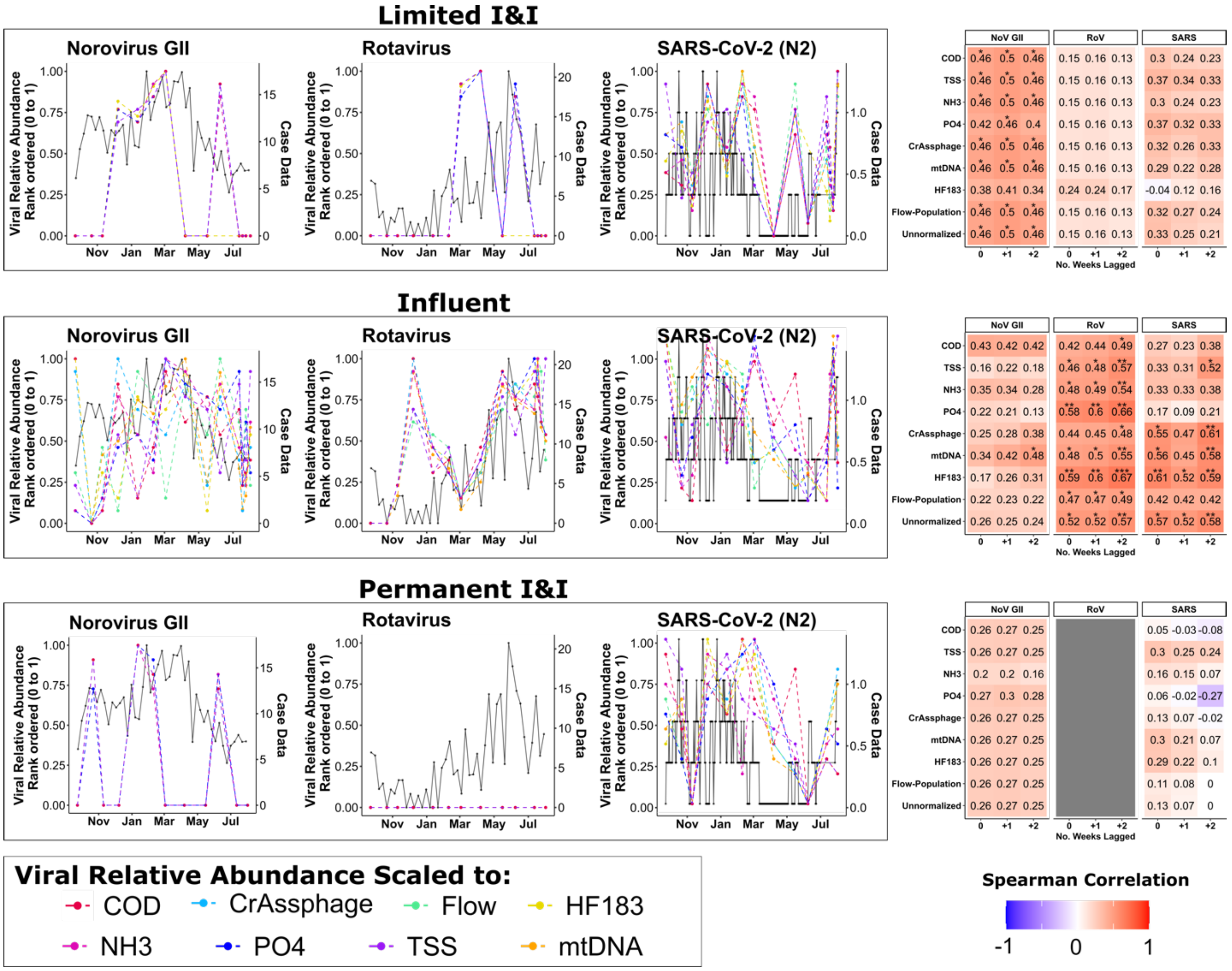

Figure S7. Relationship between normalized wastewater trends and corresponding case data. To better visualize the effect of each normalization technique on trends for infection and wastewater virus levels, we rank ordered case data and viral signal from zero to one in panel (a). Dashed lines may overlap in panel (a) and if one normalization metric is not visible this is the result of overlapping. Untransformed relative abundances were used for all Spearman correlation analyses in panel (b) as opposed to rank order scaling. Sample sizes for correlation analyses is provided in Table S8. Statistical significance for each Spearman correlation test is represented as follows: *: p≤0.10; **: p≤0.05; ***: p≤0.01; ****: p≤0.001.
